# A Hetero-Multimeric Chitinase-Containing *Plasmodium falciparum* and *Plasmodium gallinaceum* Ookinete-Secreted Protein Complex Involved in Mosquito Midgut Invasion

**DOI:** 10.3389/fcimb.2020.615343

**Published:** 2021-01-08

**Authors:** Kailash P. Patra, Hargobinder Kaur, Surendra Kumar Kolli, Jacob M. Wozniak, Judith Helena Prieto, John R. Yates, David J. Gonzalez, Chris J. Janse, Joseph M. Vinetz

**Affiliations:** ^1^ Section of Infectious Diseases, Department of Internal Medicine, Yale School of Medicine, New Haven, CT, United States; ^2^ Department of Parasitology, Leiden University Medical Center, Leiden, Netherlands; ^3^ Department of Pharmacology and the Skaggs School of Pharmacy and Pharmaceutical Sciences, University of California San Diego, La Jolla, CA, United States; ^4^ Department of Molecular Medicine, The Scripps Research Institute, La Jolla, CA, United States; ^5^ Department of Chemistry, Western Connecticut State University, Danbury, CT, United States

**Keywords:** *Plasmodium*, chitinase, complex, invasion, malaria-transmission

## Abstract

Malaria parasites are transmitted by *Anopheles* mosquitoes. During its life cycle in the mosquito vector the *Plasmodium* ookinete escapes the proteolytic milieu of the post-blood meal midgut by traversing the midgut wall. This process requires penetration of the chitin-containing peritrophic matrix lining the midgut epithelium, which depends in part on ookinete-secreted chitinases. *Plasmodium falciparum* ookinetes have one chitinase (PfCHT1), whereas ookinetes of the avian-infecting parasite, *P. gallinaceum*, have two, a long and a short form, PgCHT1 and PgCHT2, respectively. Published data indicates that PgCHT2 forms a high molecular weight (HMW) reduction-sensitive complex; and one binding partner is the ookinete-produced von Willebrand A-domain-containing protein, WARP. Size exclusion chromatography data reported here show that *P. gallinaceum* PgCHT2 and its ortholog, *P. falciparum* PfCHT1 are covalently-linked components of a HMW chitinase-containing complex (> 1,300 kDa). Mass spectrometry of ookinete-secreted proteins isolated using a new chitin bead pull-down method identified chitinase-associated proteins in *P. falciparum* and *P. gallinaceum* ookinete-conditioned culture media. Mass spectrometry of this complex showed the presence of several micronemal proteins including von Willebrand factor A domain-related protein (WARP), ookinete surface enolase, and secreted ookinete adhesive protein (SOAP). To test the hypothesis that ookinete-produced PfCHT1 can form a high molecular homo-multimer or, alternatively, interacts with *P. berghei* ookinete-produced proteins to produce an HMW hetero-multimer, we created chimeric *P. berghei* parasites expressing *PfCHT1* to replace *PbCHT*1, enabling the production of large numbers of PfCHT1-expressing ookinetes. We show that chimeric *P. berghei* ookinetes express monomeric PfCHT1, but a HMW complex containing *PfCHT1* is not present. A better understanding of the chitinase-containing HMW complex may enhance development of next-generation vaccines or drugs that target malaria transmission stages.

## Introduction

*Plasmodium* parasites cause human malaria, one of the deadliest vector-borne diseases, which has medical and economic impacts affecting nearly half of the world’s populations (Delves et al., 2019). **Human-infecting** *Plasmodium* parasites complete a complex life cycle that alternates between definitive female *Anopheline* mosquito and the intermediate human hosts. Despite continuous control measures to reduce transmission, malaria remains a significant public health threat in most tropical and sub-tropical countries (Mlambo et al., 2008). This parasitic human-mosquito-human journey results in more than 400,000 thousand deaths annually; mostly young children under five years of age living in sub-Saharan Africa (Murray et al., 2012; Viebig et al., 2015; Greenwood, 2017). The effectiveness of malaria control programs is deteriorating due to the emergence of drug resistance to artemisinin-based combination therapies (ACTs) - the only available regimen in malaria-endemic countries (Cui et al., 2015) - and the lack of an effective malaria vaccine. The World Health Organization’s Millennium Goals for malaria elimination are not likely to be met in the foreseeable future through existing approaches and technology (Mahmoudi and Keshavarz, 2017). The mosquito midgut gamete, zygote, and ookinete parasite stages are vulnerable developmental bottlenecks in the parasite life cycle and offer opportunities for the elimination of malaria parasites as targets for novel transmission-blocking vaccines, drugs, or transgenic mosquitoes expressing anti-parasite effector molecules (Sinden, 2015; Delves et al., 2018).

The malaria transmission cycle starts when a mosquito ingests a human host blood meal containing the sexual stages of the parasite. Gametogenesis and fertilization of male and female gametes occur within the mosquito midgut and result in zygote formation. Motile, crescent-shaped ookinetes transform from zygotes within hours, which then traverse the peritrophic matrix (Giersing et al., 2019) and midgut epithelium to continue the transmission cycle by developing into sporozoite-producing oocysts (Mlambo et al., 2008). Midgut gametocytes, gametes, zygotes, and ookinetes are targeted by transmission-blocking vaccines (TBVs) that are currently under clinical development (Duffy and Patrick Gorres, 2020). Malaria TBVs are a community-wide approach akin to seeking herd immunity and target developmentally-regulated antigens expressed during the mosquito midgut parasite stages, to interrupt the sporogonic cycle and prevent transmission from humans to mosquitoes (Langer and Vinetz, 2001). TBVs can synergize with other control and elimination strategies, such as insecticide-treated bednets, with the potential for global malaria eradication.

The key role of *Plasmodium* chitinases in enabling the sporogonic cycle has been described (Shahabuddin et al., 1993; Dessens et al., 2001; Langer et al., 2002a). The *Plasmodium* ookinete-secreted chitinase(s) are endochitinases in family 18 glycohydrolases that facilitate the parasite to dissolve the chitin (N-acetylglucosamine polymer)-containing peritrophic matrix (Giersing et al., 2019) within the mosquito midgut, *en route* to crossing the midgut epithelium (Shahabuddin et al., 1993; Shen and Jacobs-Lorena, 1998; Langer and Vinetz, 2001; Sinden, 2017a). An electron microscopy study of *P. gallinaceum* showed the ookinete focally disrupting and traversing the PM (Sieber et al., 1991). *Plasmodium* ookinete-secreted chitinases are of two types: short forms (*PfCHT1, PgCHT2*, and *PrCHT1*) which possess an N-terminal proenzyme and a C-terminal chitin-binding domain (CBD), and long-forms (*PgCHT1, PvCHT1*, and *PbCHT1*) which lack both domains (Li et al., 2005). The chitinase inhibitor, allosamidin, and anti-chitinase antibodies are known to block or reduce parasite transmission from the vertebrate host to mosquito, suggesting that the PM is a critical barrier to *Plasmodium* invasion of the midgut (Vinetz et al., 1999; Vinetz et al., 2000; Dessens et al., 2001; Takeo et al., 2009). However, details of the molecular mechanisms and macromolecular interactions that ookinetes use to invade the mosquito midgut remain unknown.

Here we demonstrate that *P. falciparum* and *P. gallinaceum* ookinetes secrete a short chitinase as a component of a hetero-multimeric, high molecular weight (HMW) chitinase-containing protein complex that includes WARP, SOAP, and ookinete-surface enolase. To investigate interactions within the complex we used the rodent malaria parasite, *P. berghei*, which is an important model system for understanding mosquito stage biology of *Plasmodium* because of its ability to be transgenically manipulated (Espinosa et al., 2017). *P. berghei* has only the long form of chitinase (*PbCHT1*), which is not part of an HMW complex. Therefore, we used a gene insertion/marker out (GIMO) system to replace *PbCHT1* with *P. falciparum* chitinase (*PfCHT1*; short form) to test the hypothesis that *PfCHT1* expressed within *P. berghei* ookinetes would use the endogenous machinery of *P. berghei* to heterologously express *PfCHT1* in a HMW complex. Using three *Plasmodium* species that infect human, avian, and rodent vertebrate hosts, respectively, we hypothesize that *Plasmodium *species-specific ookinete-secreted micronemal partner proteins play a vital role in the formation of a chitinase-containing hetero-multimeric HMW complex. The chitinase-containing HMW complex secreted by ookinetes binds to chitin with high affinity and might mediate recognition, attachment, and invasion of the mosquito midgut. Disruption of the complex could represent a novel Achilles heel for interrupting malaria transmission. These cross-species approaches to studying the ookinete invasion of the mosquito midgut may provide a new dimension to our understanding of the biology of malaria transmission biology and underpin future experimental approaches to delineate mechanisms by which the *Plasmodium* ookinete invades the mosquito midgut.

## Materials and Methods

### Animal Studies, Malaria Parasites, and Mosquitoes

All animal experiments of this study were granted with a license by Competent Authority after ethical evaluation by the Animal Experiments Committee Leiden (AVD1160020171625) and Institutional Review Board and the Institutional Animal Care and Use Committee (IACUC) of Yale University (Protocol ID: 2019-20243). All experiments were performed in accordance with the Experiments on Animals Act (Wod, 2014), which is the applicable legislation in Netherlands in accordance with the European guidelines (EU directive no. 2010/63/EU) regarding the protection of animals used for scientific purposes. Female OF1 and male Swiss Webster mice (6–7 weeks; Charles River, NL and MA, USA) were used for *P. berghei* infection. The Institutional Animal Care and Use Committee (IACUC), University of California San Diego, approved the animal protocol used for the production of *P. gallinaceum* ookinetes in 4 to 6 weeks-old White Leghorn chickens (Charles River, MA, USA).

A reference *P. berghei* ANKA parasite line (1868cl1) (www.pberghei.edu, line RMgm-1320) was used to generate the chimeric *P. berghei* lines. This reporter line does not contain a drug-selectable marker and expresses *mCherry* under the control of the constitutive *hsp70* promoter (*PBANKA*_0711900) and luciferase under the control of the constitutive *eef1α* promoter (*PBANKA*_1133400). These reporter gene expression cassettes are integrated into the neutral *p230p* gene locus (*PBANKA*_0306000). The detailed methodology for the generation of transgenic *P. berghei* parasite cell lines is given at the end of the methods section. The avian malaria parasite, *P. gallinaceum* 8A strain, was obtained from MR4 (BEI Resources, Manassas, VA, USA) to infect chickens following the approved protocol. The gametocyte producing *P. falciparum NF54* strain was obtained from MR4 and *in vitro* cultured following a standard method (Bounkeua et al., 2010).

*A. stephensi* mosquitoes were housed and maintained at 27°C and 75% relative humidity under a 12-h light/dark cycle at Leiden University Medical Center. For *P. berghei* infections, mosquitoes were fed one time (day 0) on anesthetized *P. berghei* infected mice and kept within a 21°C and 80% humidity incubator and dissected 10-12 days after blood feeding for midgut and oocyst counts.

### *In Vitro* Production of *Plasmodium gallinaceum, Plasmodium falciparum*, and *Plasmodium berghei* Ookinetes

The avian malaria parasite, *P. gallinaceum* 8A strain passage 1 (blood from the chicken that was infected through the bites of infective mosquitoes), was used to infect 4 to 6 weeks-old White Leghorn chickens. At 5%–10% parasitemia, heparinized blood was collected by cardiac puncture and used immediately for *in vitro* production of ookinetes following a published method (Patra and Vinetz, 2012). Purified zygotes stages were incubated in serum-free medium (M199 containing 0.2% glucose, 2 mM L-glutamine, 50 units of penicillin, and 50 µg of streptomycin/ml) at 26°C for 36 h to allow transformation into mature ookinetes. Cultures with above 50% ookinete transformation were centrifuged (4,000 rpm, 10 min), and supernatants were collected for further use.

The *P. falciparum* NF54 strain was used for *in vitro* gametocyte and ookinete production per a published method with slight modifications (Bounkeua et al., 2010). After the fertilization step, the zygote pellets were washed three times with serum-free ookinete medium (M199 medium containing 0.2% glucose, 2 mM L-glutamine, 50 units of penicillin, and 50 µg of streptomycin/ml) and incubated in 20 ml of serum-free M199 medium at 28°C for 48 h in a slow-shaking incubator. Cultures with >30% ookinete transformation were used for biochemical studies. Human serum and other animal serum-containing media show high chitinase activity, and we therefore developed a serum free *P. falciparum* ookinete culture method for these studies, which needs improvement to achieve higher ookinete transformation.

*P. berghei* ookinetes were obtained as described (Janse et al., 1985; Janse and Waters, 1995). The gametocyte conversion rate is defined as the percentage of ring-forms that develop into gametocytes in standard synchronized *in vivo* infections in mice (done in triplicate). *In vitro* ookinete formation assays were performed following published methods using gametocyte-enriched blood collected from mice (Janse et al., 1985). Briefly, infected blood from ~3 to 5 mice containing gametocytes was mixed in standard ookinete culture medium and cultures were incubated for 18–24 h at 21°C –22°C. Between 12 and 20 min after the activation of gametocytes the number of exflagellating male gametocytes was determined by counting exflagellating males (in triplicate) in a Bürker cell chamber. The fertilization rate (ookinete conversion rate), defined as the percentage of female gametes that develop into zygotes or ookinetes, was determined (in triplicate) by counting female gametes and zygotes/ookinetes in Giemsa-stained blood smears at 18–24 h after *in vitro* induction of gamete formation. The ookinete cultures were further centrifuged as mentioned above and passed through 0.22 µm filters (50 ml Steriflip, EMD Millipore, MT). In the case of *P. berghei* infection of *A. stephensi* mosquitoes, collection and counting of oocyst (at day 12–14 post feeding) were performed as described (Sinden, 1997; Janse et al., 2006; Othman et al., 2018).

### Size Exclusion HPLC Fractionation of Ookinete Culture Supernatants and Analysis of Chitinase Activity

Approximately 50 ml of *P. gallinaceum* ookinete culture supernatant was concentrated using a 10 kDa cut off Centricon device, EMD Millipore, MT to 1 ml final volume. Chitinase activity of the concentrated sample was tested before loading onto a silica-based SEC column (TSKgel G2000SW, Tosoh Biosciences LLC, King of Prussia, PA), and HPLC was performed using a Beckman Gold 500 instrument and autoclaved PBS (pH 7.4). Approximately 100 µl of each fraction (total 0.5 ml volume/1 min) was analyzed in duplicate for the presence of chitinase activity that showed two distinct activity peaks. Gel filtration standards (Bio-rad, Hercules, CA) were analyzed in similar conditions and the retention times of the protein sizes were determined. Similarly, concentrated serum-free ookinete culture supernatant of *P. falciparum* was subjected to HPLC following the condition for *P. gallinaceum*; however, it showed low activity in the fractions due to the sample dilutions, but showed two distinct peaks when fractions were coated on ELISA plated and probed with 1C3, anti-PfCHT1 monoclonal antibodies (data not shown). To further resolve the HMW complex and determine the approximate mass, subjected samples were used for high-resolution chromatography, immunodetection, and estimation of the chitinase complex in both *P. falciparum* and *P. gallinaceum.*

### Size Exclusion Chromatography for Estimation of the Native Chitinase Complex Molecular Weight

Ookinete culture supernatants (*P. gallinaceum* and *P. falciparum*) were passed through 0.22 µm spin columns and loaded separately onto gel filtration columns (Superose-6, 10/300, MW separation range 5–5,000 kDa of globular proteins) in PBS, pH 7.4, and fractions (0.5 ml/min) were collected on ice. Each fraction was coated onto duplicate wells of an ELISA plate and probed with anti-chitinase mAb (1C3) followed by goat-anti-mouse IgG-HRPO conjugate and BCIP/NBT substrate addition to detect the chitinase in the fraction coated wells. The absorbance optical density (OD) values were plotted to determine the peak vs. retention time of both *P. falciparum* and *P. gallinaceum* ookinete culture supernatants. Blue Dextran was used to estimate the void (V_o_, 2 min retention time) volume of the column and commercially available gel filtration standards (17–670 kDa) were run under the same conditions and retention times were noted. We used a standard method to calculate the molecular weight based on the retention time of the known standards used to estimate the partition coefficient (K_av_ = V_e_ – V_0_/V_t_ – V_0_) and a standard calibration curve (Panel C) was plotted; where V_0_ is the void volume, V_e_ is the elution volume of the sample, and V_t_ is total column volume. The molecular weights of the fractions containing the peak chitinase were estimated from the equation and are presented in the **Supplementary Data Sheet 1**.

### Chitinase Activity and Chitin Affinity Pull-Down of the Chitinase Complex From Ookinete Culture Supernatants, SDS-PAGE, and Western Immunoblot Analysis

Chitinase activity of the ookinete supernatants from the three *Plasmodium* species was tested by adding chitinase substrate (4-methylumbelliferyl-N, N′, N″-β-D-triacetylchitotrioside; Sigma), and the linear increase in the enzymatic activity was determined by kinetic fluorescence detection (Gemini EM, Molecular Devices LLC, CA; excitation, 365 nm; emission, 450 nm) as described (Li et al., 2005). Chitin beads (Catalog, S6651S, New England Biolabs, MA) were washed three times with PBS (pH 7.4) and made into a 50% slurry with PBS. To 10 ml of 0.22 µm filtered ookinete culture supernatant, 200 µl of 50% chitin bead slurry was added and incubated at 4°C overnight on a slow rocker. The tubes were centrifuged (2,300 rpm, 5 min) and protein bound chitin beads were washed three times with PBS or PBST (0.1% Tween 20) and SDS-sample buffer was added directly to the beads. The samples were then heated for 3 min at 70°C and subjected to SDS/PAGE under reducing (β-mercaptoethanol) and non-reducing conditions. Primary antibody incubation was done with 5% nonfat dry milk in PBS containing (0.1% Tween 20) (PBST) overnight at 4°C. Secondary antibody controls were run with each blot, under non-reducing and reducing conditions. For the detection of bound primary antibodies, the membrane was washed three times with PBST and incubated with alkaline phosphatase-conjugated goat anti-mouse IgG (H+L) antibody (1:2500) (KPL, USA) in 5% nonfat dry milk in PBS containing (0.1% Tween 20) (PBST) at RT for 1 h. The blot was then developed with a 5-bromo-4-chloro-3-indolyl phosphate (BCIP)/nitro blue tetrazolium (NBT) solution (KPL, MD, USA). For *P. gallinaceum*, one SDS-PAGE gel was stained with silver nitrate (Silver Quest kit, Thermo Fisher Scientific) and the other was subjected to Western immunoblot analysis. Membranes containing the *P. gallinaceum* and *P. falciparum* pull-down samples were probed with the anti-chitinase active site peptide mouse polyclonal antibody, B993 (Langer et al., 2002a), and anti-*PfCHT1* (1C3) monoclonal antibodies, as reported (Langer et al., 2002b). For the three *P. berghei* parasite lines (*Pb-PfCHT1(r)*, *PbCHT1 (WT)*, and *PbΔCHT1*), membranes run under both conditions were probed using anti-*PfCHT1* monoclonal (1C3, 25 μg/ml) and anti-*PbCHT1* polyclonal antibodies (1:2000 dilutions) as described in the figure legends.

### Mass Spectrometry Analysis of the Ookinete Secreted Chitinase Complex

To identify proteins associated with *P. gallinaceum* chitinases (*PgCHT1* and *PgCHT2*), washed protein-bound beads were subjected directly to enzymatic digestions, and MudPIT analysis and the collected MS/MS spectra were analyzed as described (Patra et al., 2008). The MS/MS spectra were verified against chicken (*Gallus gallus*) protein entries, and manually compared with a list of common contaminants (e.g., human keratin and trypsin). The final list of peptides sequences was used for BLAST searches against the PlasmoDB databases (PlasmoDB.org) to identify proteins and their function. For *P. falciparum*, the chitinase complex bound chitin beads were washed three times with PBS and submitted for mass spectrometry* *analysis. The chitinase complex-bound chitin beads were suspended in 8M urea to denature and detach the bound proteins. Following resuspension, proteins were reduced, alkylated, and digested with LysC and trypsin. Tryptic peptides were desalted *via* Sep-Pak (Waters, MA, USA) and dried in a speed vacuum overnight. The digested peptides were quantified using the Pierce quantitative fluorometric peptide assay (Thermo Fisher Scientific, Carlsbad, CA). The digested peptides were analyzed on an Orbitrap Fusion Mass Spectrometer with an in-line Easy-nLC 1000. Samples were loaded onto the column (pressure 500 bar and eluted over 85 min with a linear gradient of 11%–30% acetonitrile in 0.125% formic acid). Nanospray ionization was achieved by applying 2000V through the stainless-steel T and the inlet of the column. MS1 was performed to select precursors for identification, peptides were fragmented to identify their amino acid sequence at the MS2 level, and the acquired spectra was analyzed against the *P. falciparum* (strain 3D7) reference proteome (UniProt) using the SEQUEST search algorithm (Eng et al., 1994). Search parameters included the dynamic oxidation of methionine and the static carbamidomethylation of cysteine (modifications that arise from sample preparation). The data was filtered to a 1% false discovery rate at both the peptide and protein levels using a reverse database search.

For *P. berghei*, mass spectrometry analysis of the chitin bound chitinase and other proteins were performed at the W.M. Keck Biotechnology Resource Laboratory, Yale University. Briefly, the sample was washed four times; first with 60% acetonitrile containing 0.1%TFA and then with 5% acetic acid, then with 50% H_2_O/50% acetonitrile, followed by 50% CH_3_CN/50 mM NH_4_HCO_3_. A final wash with 50% CH_3_CN/10 mM NH_4_HCO_3_ was given and gel pieces were completely dried prior to removal of the wash solution. Trypsin digestion was carried out by incubating the sample at 37°C for 18 h and the samples were stored at -20°C until analysis (Ghosh et al., 2019). The trypsin digested proteins products were fractionated using LC-MS/MS. The Mascot algorithm was used for all MS/MS spectra searches (Hirosawa et al., 1993) (Matrix Science, London, UK; version Mascot in Proteome Discoverer 2.2.0.388) and Mascot was set up to search the *PbergANKA*_PDB11 and Plasmo_*Pfal3D7* databases. Protein identification criteria were the match of 2 or more peptides with 90% peptide threshold, a Decoy FDR of 0.5%, and a mascot ion score of >30.

### Site-Directed Mutagenesis of Conserved Cysteines of Plasmodium gallinaceum Chitinase (PgCHT1) and Chitin Binding Affinity Analysis of rPgCHT1 and rPfCHT1

*PgCHT1* putative chitinase binding domain mutants were constructed using a QuickChange Multi Site-Directed Mutagenesis Kit (Agilent Technology, CA). The expression construct *PgCHT1*- pET32b plasmid DNA (Langer et al., 2000) was used as a template to amplify the long form of *P. gallinaceum* chitinase (*PgCHT1*) which was already synthesized in *E. coli*-preferred codons. The primers were designed to modify bases at positions 1,355 bp (TGC to TCC), 1,502 bp (TGC to TCC), and 1,670 bp (TGT to TCT) of the plasmid construct to mutate three cysteine (C) residues to serine (S). These three cysteines are conserved across the long form of *Plasmodium* chitinases of different *Plasmodium* species and are located in the putative chitin-binding domain at the C-terminal end of the *P. gallinaceum* chitinase, *PgCHT1* (Li et al., 2005). The mutant *PgCHT1*- pET32b plasmid constructs (1 and 2, **Figure 6**) were confirmed by Sanger sequencing before expressing as recombinant protein. We also synthesized *PfCHT1*- pET-32b expression plasmid (Gene Universal, Newark,DE) in *E. coli* preferred codons to express rPfCHT1 in *E.coli*.

The mutant plasmids (1 and 2 mutant-*PgCHT1*-PET32b) and wild type (*PgCHT2*-pET32 and *PfCHT1*-pET32b) were transformed into SHuffle T7 Express Competent *E. coli* cells (New England Biolabs, MA). Small scale protein expression was carried out as described (Li et al., 2005). Bacterial pellets from 1 ml of IPTG (isopropyl-ß-D-1-thiogalactopyranoside) induced cultures were suspended in 500 µl of PBS and sonicated (Sonicator 3000, Misonix, CT) using a microprobe for 3 min (10 s on and 10 s off mode) on ice. Similarly, recombinant *E. coli* expressing *PfCHT1* were lysed using mild detergent at room temperature and a fraction (1 ml) of the lysate was used for the chitin pull-down assays and the rest of the soluble fraction used for the Ni-NTA purification using HisTrap HP columns (Cytiva, Marlborough, MA).

For chitin binding assays, lysates from rPgCHT, rPgCHT1 mutants and r*PfCHT1* were centrifuged and supernatants were incubated in a rotor shaker for 30 min with washed chitin beads. Further centrifugation was performed to wash the chitin bead pellet three times with PBS with or without 1% Triton X-100 for *PgCHT1* and 0.2%–1% Triton X-100 for *PfCHT1*. After the final washing sample buffer was added directly to the protein-bound chitin beads, boiled for 5 min, and analyzed by SDS-PAGE and western immunoblot. The membrane was probed with anti-thioredoxin monoclonal antibodies (Abcam, MA) to detect bound recombinant *PgCHT1* proteins in the wild types and mutants (1 and 2) in which all the three conserved cysteines were modified in the chitin binding domain. For *PfCHT1*, the membranes were incubated with 1C3 monoclonal antibody to detect *PfCHT1* in the ookinete culture supernatant pull-downs.

### Generation of DNA Constructs and Genotyping of Chimeric *Plasmodium berghei* Expressing *PfCHT1*

The detailed sequence and names of the primers used for PCR and genotyping are listed (**Supplementary Table 1**). A two-step gene insertion/marker out (GIMO) transfection protocol was used to generate chimeric parasites in which the *PbCHT1* coding sequence (CDS; PBANKA_0800500) was replaced by the CDS of *PfCHT1* (PF3D7_1252200). In the first step we deleted the *PbCHT1* CDS by replacing it with the positive-negative selectable marker (SM) to create a *P. berghei CHT1* deletion GIMO line, *PbΔCHT1.* To do this, we generated DNA construct pL2321, which is based on the standard GIMO DNA construct pL0034. This construct contains the positive-negative human dihydrofolate reductase: yeast cytosine deaminase, and a uridyl phosphoribosyl transferase (h*dhfr: yfcu*) selection marker (SM) cassette. The 5’ and 3’ targeting regions of *PbCHT1* were amplified from *P. berghei* ANKA genomic DNA using the primers 9450/9451 and 9452/9453. Fragments were digested with ApaI/SacII and KpnI/NotI, respectively, and ligated into vector pL0034 to obtain pL2321. For transfection, pL2321 was linearized with ApaI/NotI and the linear construct was introduced into parasites of the *P. berghei* ANKA reference line 1868cl1 using standard transfection methods (Janse et al., 2006). Transfected parasites (exp. 3152) were positively selected in mice by providing pyrimethamine in the drinking water. Transfected parasites were cloned by limiting dilution, resulting in cloned line ***PbΔCHT1*** (line 3152cl1). The correct deletion of *PbCHT1* was confirmed by diagnostic PCR analysis of genomic DNA (gDNA) and Southern analysis of pulsed-field gel (PFG)-separated chromosomes. In the second step, we replaced the positive-negative SM cassette in *PbΔCHT1* with the *PfCHT1* CDS by GIMO transfection to create the *P. berghei* chimeric *Pb-PfCHT1*(r) replacement line. This was achieved by modifying the construct used in the first step (pL2321); specifically, the h*dfhr*: yfcu SM cassette was removed and replaced with the *Pfcht1* CDS sequence, generating plasmid pL2322. The *PfCHT1* CDS was amplified from the DNA of *P. falciparum* NF54 (PF3D7_1252200) using the primers 9454/9455. *PfCHT1* CDS fragment was digested with SacII/KpnI and ligated into vector pL2321 to obtain pL2322. The pL2322 construct was sequenced to ensure that there were no mutations in the *PfCHT1* CDS. The construct was linearized using ApaI and NotI restriction enzymes outside the 5’ and 3’ targeting regions before transfection. The construct was used to transfect parasites of *PbΔCHT1* (line 3152cl1) using standard methods of GIMO transfection (Lin et al., 2011; Salman et al., 2015). Transfected parasites (exp 3165) were negatively selected in mice by providing 5-fluorocytosine (5-FC) in the drinking water. Negative selection results in the selection of chimeric parasites where the h*dhfr*::y*fcu* SM in the *cht1* locus of *PbΔCHT1* is replaced by the CDS of *PfCHT1.* Selected chimeric parasites were cloned by limiting dilution resulting in the cloned line 3165cl1 (*Pb-PfCHT1*(r)). Correct integration of the constructs into the genome of *Pb-PfCHT1*(r) was analyzed by diagnostic PCR analysis of gDNA and Southern analysis of PFG-separated chromosomes as described (Janse et al., 2006). The PFG-separated chromosomes were hybridized with a mixture of two probes: a probe recognizing the h*dhfr* gene and an ~800 bp fragment of the 5′UTR of PBANKA_0508000 located on chromosome 5. This method creates chimeric gene replacement *P. berghei* parasites that lack the *Pbcht1* CDS but possess the *PfCHT1* CDS under the control of the *PbCHT1* 3’- and 5’-UTR regulatory sequences.

## Results

### *In Vitro* Production of *Plasmodium gallinaceum*, *Plasmodium falciparum*, and *Plasmodium berghei* Ookinetes in Serum-Free and 5% Serum-Containing Media

**Figure 1:**
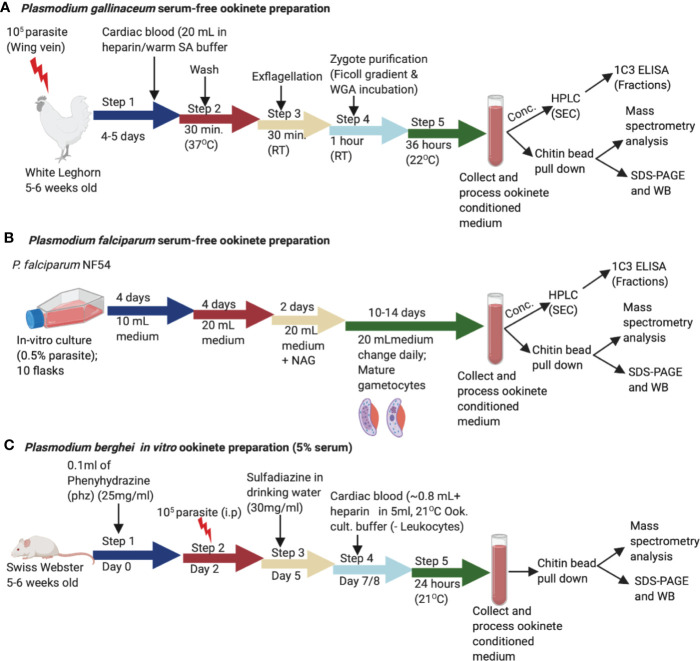
Schematic representation of the *in vitro* production of *Plasmodium gallinaceum* and *Plasmodium falciparum* ookinetes in serum free media and *Plasmodium berghei* lines (*Pb-PfCHT1* (r)*, PbCHT1* (WT), and *PbΔCHT1*). **(A)** Blood samples were collected into suspended animation buffer from *P. gallinaceum* infected White Leghorn chickens, centrifuged, and process as described in the methods section. For transformation to mature ookinete stages, purified zygote pellets were washed twice with ookinete culture serum free medium and incubated in the same media at 26°C for 36 h. The culture supernatants were characterized with a chitin bead pull-down assay, mass spectrometry, size exclusion fractionation, and immunological detections of a high molecular weight complex. **(B)** Synchronized *P. falciparum* NF54 cultures were seeded at 0.5% parasitemia with 5% hematocrit in 10 ml complete growth medium and cultured for gametocyte production as described in the methods section. The gametocyte cultures were pelleted, added to exflagellation media, and incubated for 30 min followed by centrifugation. The pellet was washed three times with serum free ookinete media and incubated in 20 ml of serum free ookinete media at 28°C for 48 h in a shaker incubator. The ookinete transformations were determined by examining Giemsa-stained thin smear slides. The culture supernatant was passed through a 0.22 µm filter, incubated with chitin beads to pull down the chitinase complex, and characterized as described above for *P. gallinaceum*. **(C)** The sulfadiazine method was used for the preparation of gametocyte-enriched blood from asynchronous infections in mice. Briefly, mice were treated with 0.1 ml phenylhydrazine (phz) to induce reticulocytosis and infected with 10^7^ erythrocytes (*Pb-PfCHT1* (r)*, PbCHT1* (WT), and *PbΔCHT1* lines). After the sulfadiazine treatment (30 mg/ml) on day 5, the mice were sacrificed and diluted in ookinete culture medium and leukocytes were removed through a Plasmodipur leukocyte filter. The *in vitro* cultures were further incubated at 21°C for 24 h. The culture supernatants were stored at –20°C for further biochemical and qualitative assays.

**Figure 2:**
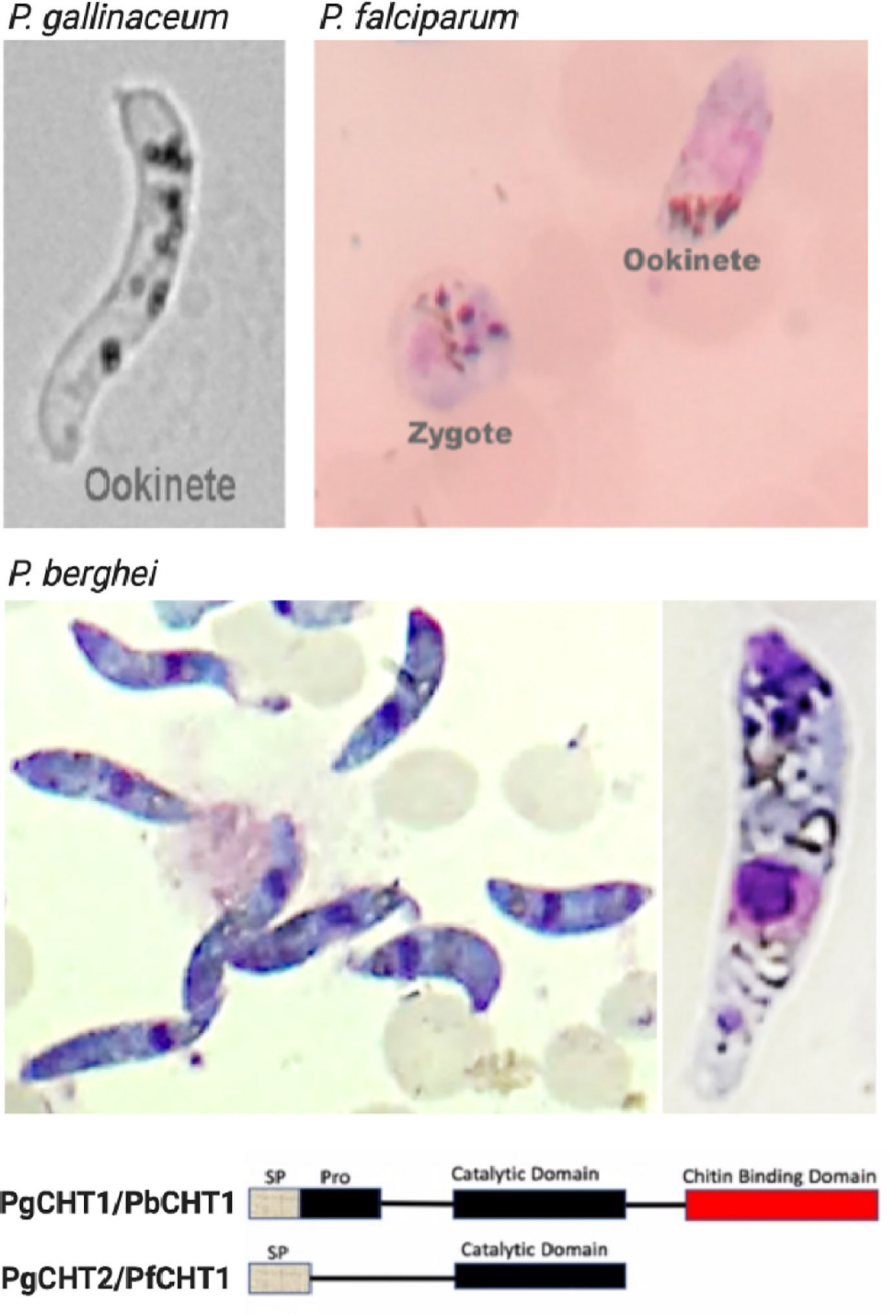
Light microscopy images of *Plasmodium gallinaceum* and *Plasmodium falciparum* ookinetes produced *in vitro* using serum-free media, and *P. berghei* ookinetes in serum-containing medium. The lower panel shows the structural feature of the two types (long and short forms) of chitinases seen in *Plasmodium*. The characteristic banana shaped *P. gallinaceum* ookinete under bright field microscopy. This species possesses long and short form chitinases (*PgCHT1*, long form with chitin binding domain and *PgCHT2*, short form without CBD). Giemsa-stained smear of *P. falciparum* ookinete culture shows ookinete and zygote stages which are difficult to differentiate under bright field. The *P. falciparum* genome has only the short form (*PfCHT1*) of chitinase which lacks a chitin binding domain and is the ortholog of *PgCHT1*. *P. bergehi* ookinete production required serum and has only the long form chitinase, *PbCHT1.* The images were taken at 1000x magnification with a cell phone camera.

Ookinetes were transformed from zygotes using serum free M199 medium for the *P. gallinaceum* 8A and *P. falciparum* NF54 lines (**Figures 1A, B**) and RPMI 1640 with 5% serum for *P. berghei* parasite lines (*Pb-PfCHT1 (r)*, *PbCHT1* (WT), and *PbΔCHT1*) (**Figure 1C**). *P. gallinaceum* preparations routinely resulted in > 50% ookinete transformation in serum-free medium. Mature ookinetes were accurately distinguished from gametocytes using light microscopy (**Figure 2**); specifically, ookinetes were identified as extracellular and with banana-shaped morphologies clearly distinguishable from gametocytes. As confirmation anti-chitinase mouse monoclonal antibodies (1C3) showed apical fluorescence on the *P. gallinaceum* ookinete stages as described [7].

Published methods describe the use of 10%–20% human serum or Albumax in culture medium for the *in vitro* production of *P. falciparum* ookinetes [10-13]. Serum- or Albumax-containing media have variable levels of chitinase activity (**Supplementary Figure 1**) that remains partially active after heat inactivation (58°C for 30–60 min), which introduced potential analytical limitations into our system. Batch adsorbing with chitin beads depletes human serum chitinase activity from human serum-containing ookinete culture media, as determined using the 4-methylumbelliferone chitotrioside substrate-based enzymatic assay (**Supplementary Figure 1**). Despite the absence of observable enzymatic activity in the culture medium, a faint band representing the human serum chitinase (approximately a band at 60 kDa reacting with anti-chitinase active site peptide antibodies) was observed by western immunoblot (data not shown). Nonetheless, *in vitro* production of mature *P. falciparum* ookinetes was improved in terms of the ability to identify parasite-specific chitinase activity. Further, a modified method for the *in vitro* culture of *P. falciparum* ookinetes in serum-free medium was developed (**Figure 1**). Enumeration of ookinetes was done using Giemsa-stained slides examined by light microscopy (**Figure 2**). The ookinete transformation efficiency was estimated at 20%–30% using serum-free M199 medium and specific modifications including incubation in a shaker incubator (60 rpm) at 28°C temperature for 48 h. The concentrated *P. falciparum* ookinete culture supernatants demonstrated detectable enzymatic activity by standard chitinase assay and were used for further studies.

*In vitro* production of *P. berghei* ookinetes for the three parasitic cell lines *Pb-PfCHT1*(r), *PbCHT1* (WT) and *PbΔCHT1* was carried out using an established protocol. The parasites showed normal asexual blood stage multiplication in mice (data not shown). In addition, *PbΔCHT1* and *Pb-PfCHT1*(r) parasites produced gametocytes and mature ookinetes *in vitro* comparable to wild type *P. berghei* (**Table 1**) with greater than 50% of the ookinete transformation rate. The fully mature Giemsa-stained *P. berghei* ookinetes are further viewed under the microscope, where the blunt apical and tapered posterior end with the presence of the nucleus can be visualized (**Figure 2**). *PbCHT1 (WT)*, *PbΔCHT1*, and *Pb-PfCHT1*(r) lines were passed through *A. stephensi* mosquitoes; and all lines produced oocysts with numbers in the expected range for *A. stephensi* mosquitoes directly fed on wild type *P. berghei* ANKA parasites infected mice (**Table 1**). This result shows that the *PbΔCHT1* formed oocysts normally in the mosquito midgut; head-to-head comparisons of WT vs. *PbΔCHT1* were not done. Earlier, membrane feeding of *PbCHT1 KO* resulted in a 30%–90% reduction of oocysts, with mean oocyst ranges from 25 to 63 oocysts/gut in PbCHT1-KO line (Dessens et al., 2001), suggesting that *P. berghei* ookinetes escape the midgut early, before the peritrophic matrix fully forms in *An. stephensi*.

**Table 1:** Gametocyte, ookinete, and oocyst production of *PbΔCHT1* (3152cl1) and *Pb-PfCHT1(r)* (3165cl1) transgenic lines in comparison to wild type strains (WT).

Mutant	Gametocyte production (%)*^a^*mean (s.d.)	Male exflagellation rate (%)*^b^*mean (s.d.)	Ookinete production (%)*^c^*mean (s.d.)	Number of oocysts per mosquito*^d^*mean (s.d.)
***PbΔCHT1* (3152cl1)**	18.3 (2.0), n=3	79 (2.4), n=3	80.3 (3.7), n=3	174.6 (104.0), n=21
***Pb-PfCHT1(r)* (3165cl1)**	20.7 (1.7), n=3	89.6 (2.5), n=3	62.5 (6.6), n=3	164.3 (91.9), n=16
**WT** *^e^*	15-25, n>10	65-95, n>10	50-90, n>10	75-249, n>10

^a^The mean percentage of blood stage parasites developing into gametocytes in vivo.

^b^The mean percentage of males that exflagellate in vitro 12–15 min after activation.

^c^The mean percentage of female gametes developing into mature ookinetes in vitro 16–18 h after activation of gametocytes.

^d^The mean number of oocysts per mosquito (days 12–13).

^e^Wild type: reference P. berghei ANKA reporter line 1868cl1.

s.d., standard deviation.

*P. gallinaceum, P. falciparum*, and *P. berghei* (*Pb-PfCHT1*(r) and *PbCHT1* (WT)) ookinete culture supernatants demonstrated detectable chitinase enzymatic activity (data not shown), as measured by a fluorometric assay using 4-methyl-umbelliferyl-N, N′, N″-β-D-triacetyl-chitotrioside (4MU) as substrate. This result suggests that the ookinete-secreted chitinases are enzymatically active and thus these preparations were used for further studies. We did not observed chitinase activity in the ookinete supernatants of PbΔCHT1 parasite lines, despite the observation of mature ookinetes.

### Identification of a *Plasmodium gallinaceum* and *Plasmodium falciparum* Ookinete-Secreted High Molecular Weight Chitinase-Containing Complex

Published data have shown that *P. gallinaceum* produces two forms of chitinase that are distinguished by SDS-PAGE and immunoblot analyses and physical characteristics as determined *via* strong anion exchange chromatography; specifically, a long form, with proenzyme and chitin binding domains, and a short form that contains the core TIM-barrel catalytic domain, but which lacks proenzyme and chitin-binding domains (Vinetz et al., 2000; Li et al., 2005). Western immuno-blot analysis following anion exchange chromatography of *P. gallinaceum* ookinete culture supernatant had shown a reduction-sensitive, high molecular mass (>200 kDa) chitinase-containing band, which was subsequently found to contain covalently bound WARP (Vinetz et al., 2000; Vinetz, 2005), but otherwise was not further characterized.

We further characterized the *P. gallinaceum* chitinase-containing high molecular mass complex by subjecting concentrated ookinete culture supernatants to HPLC size exclusion chromatography (TSKgel G2000SW), and fractions (0.5 ml/min) were collected and analyzed for chitinase activity (**Supplementary Figure 2**). We observed two peaks of *P. gallinaceum* chitinase activity, one just before the retention time of 11.5 min and the other at 14.0 min (**Supplementary Figure 2**). To determine whether the sole chitinase of *P. falciparum* (PfCHT1), the ortholog of the second, short form *P. gallinaceum* chitinase, PgCHT2, binds to chitin, concentrated *P. falciparum* ookinete culture medium was analyzed by HPLC as was done for *P. gallinaceum*. Because the protein yields of *P. falciparum* ookinete cultures are lower than those of *P. gallinaceum* ookinete cultures, we could not detect chitinase activity in the fractionated samples. Therefore, we used high resolution size exclusion chromatography and ELISA to detect chitinase-containing fractions, using the anti-chitinase monoclonal antibody, 1C3, for detection (Langer et al., 2002a).

### Size Exclusion Chromatography of *Plasmodium gallinaceum* and *Plasmodium falciparum* Ookinete Culture Supernatants

**Figure 3:**
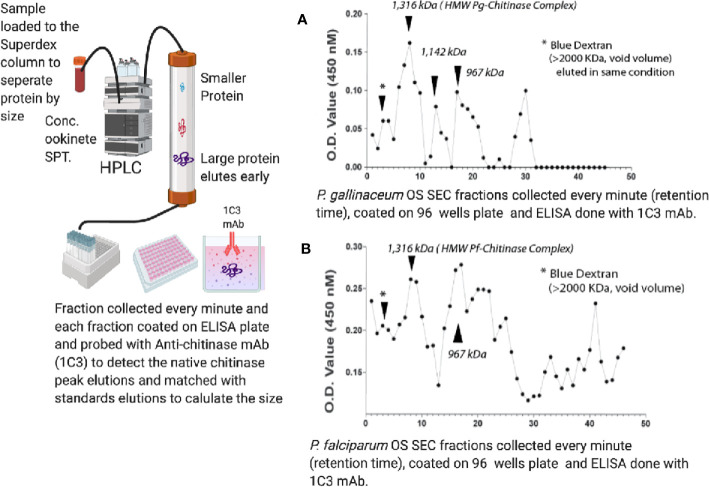
Size exclusion chromatography estimation of the molecular mass of the ookinete-secreted native chitinase complex in ookinete culture supernatants of *P. falciparum* **(A)** and *P. gallinaceum* **(B)**. Concentrated ookinete culture supernatants from *P. gallinaceum* and *P. falciparum* were loaded separately onto a gel filtration column (Superose-6, 10/300, MW separation range 5–5,000 kDa of globular proteins) in PBS, pH 7.4, and fractions (0.5 ml/min) were collected on ice. Each fraction was coated onto duplicate wells of an ELISA plate, probed with anti-chitinase mAb (1C3), followed by goat-anti-mouse IgG-horse radish peroxidase-conjugate and 5-bromo-4-chloro-3-indolyl phosphate (BCIP)/nitro blue tetrazolium (NBT) substrate. Absorbance/optical density (OD) values were plotted to determine the peak versus retention times of both *P. falciparum* ookinete **(A)** and *P. gallinaceum* ookinete culture supernatants **(B)**. Dextran Blue was used to estimate the void (V_o_) volume of the column and commercially available gel-filtration standards (17–670 kDa) were run in the same conditions, with retention times and chromatogram shown in **Supplementary Figure 3**. The retention time of the standards were used to estimate the partition coefficient (K_av_ = V_e_ – V_0_/V_t_ – V_0_) and a standard calibration curve (**Supplementary Figure 3**) was plotted. V_0_ is void volume, V_e_ is the elution volume of the sample, and V_t_ is the total column volume. The molecular weight of the fraction containing the peak chitinase was estimated from the equation, and details are presented in the table below and calculation in **Supplementary Table 3**. Blue dextran (void volume) had a 2 min retention time, whereas chitinase-containing fractions of *P. gallinaceum* and *P. falciparum* ookinete culture supernatants showed an early peak at 8 min, which corresponds to a protein MW of roughly 1,300 MDa, yielding the estimated size of the chitinase complex in native forms.

To estimate the mass of the ookinete-secreted, short chitinase-containing high molecular complex, we followed standard methods of high-resolution gel filtration chromatography (Superose-6, 10/300, MW separation range 5–5,000 kDa of globular proteins) and fractions (0.5 ml/min) were collected on ice. Each fraction was coated onto duplicate wells of an ELISA plate, and the presence of the short-form chitinase was detected by anti-chitinase mAb (1C3) [which reacts with *PfCHT1* and *PgCHT2* but not *PgCHT1*; (Langer et al., 2002a)] and absorbance/optical density (OD) values were plotted to determine the peak vs. retention times of both *P. falciparum* (**Figure 3A**) and *P. gallinaceum* (**Figure 3B**) ookinete culture supernatants. Blue Dextran was used to estimate the void (Vo) volume of the column, and commercially available gel-filtration standards were run in the same conditions (retention times and chromatograms are shown in **Supplementary Data File 1**). The approximate size of the peak was estimated by calculating the partition coefficient (K_av_) and a standard calibration curve was plotted to estimate the molecular weight (Ohno et al., 1986) following the manufacturer’s instructions (https://cdn.cytivalifesciences.com). The molecular weight of the fraction containing the peak chitinase was estimated from the equation presented (**Supplementary Data File 1**). Blue dextran was used as a void volume determination and had a 2 min retention time, whereas chitinase containing *P. gallinaceum* and *P. falciparum* ookinete culture supernatants showed early peaks at 8 min, which correspondence to an estimated MW of 1,300 kDa, approximately the predicted size of the chitinase complex in native forms. These observations indicate that the first 1C3-positive peak represents the chitinase complex and the second peak represents free monomeric chitinase present in the ookinete culture supernatants.

### Identification of Chitinase-Binding Partners by Chitin Bead Affinity Pull-Down of *Plasmodium gallinaceum*, *Plasmodium falciparum*, and *Plasmodium berghei* Chitinases From the Ookinete Secretome

*P. gallinaceum*, *P. falciparum*, and *P. berghei* ookinete culture supernatants were centrifuged, concentrated, and passed through 0.22 µm filters prior to chitin bead affinity pull-down experiments. Immuno-precipitation (IP) using anti-chitinase antibodies (mAb-1C3, rabbit anti-*PgCHT1* polyclonal antibodies, and anti-active site peptide antibodies) had failed to pull down chitinase(s) or chitinase complexes from ookinete culture supernatants. Therefore, an alternative approach was used in which chitin beads were added directly to the 0.22 µm filtered ookinete culture supernatant to allow the parasite chitinase(s) to interact directly with the chitin. The chitin bound proteins were then pelleted and washed to remove non-specific binding. SDS sample buffer was then added directly to the pelleted chitin beads, which were subjected to SDS-PAGE and western immunoblots performed under both non-reducing and reducing conditions.

**Figure 4:**
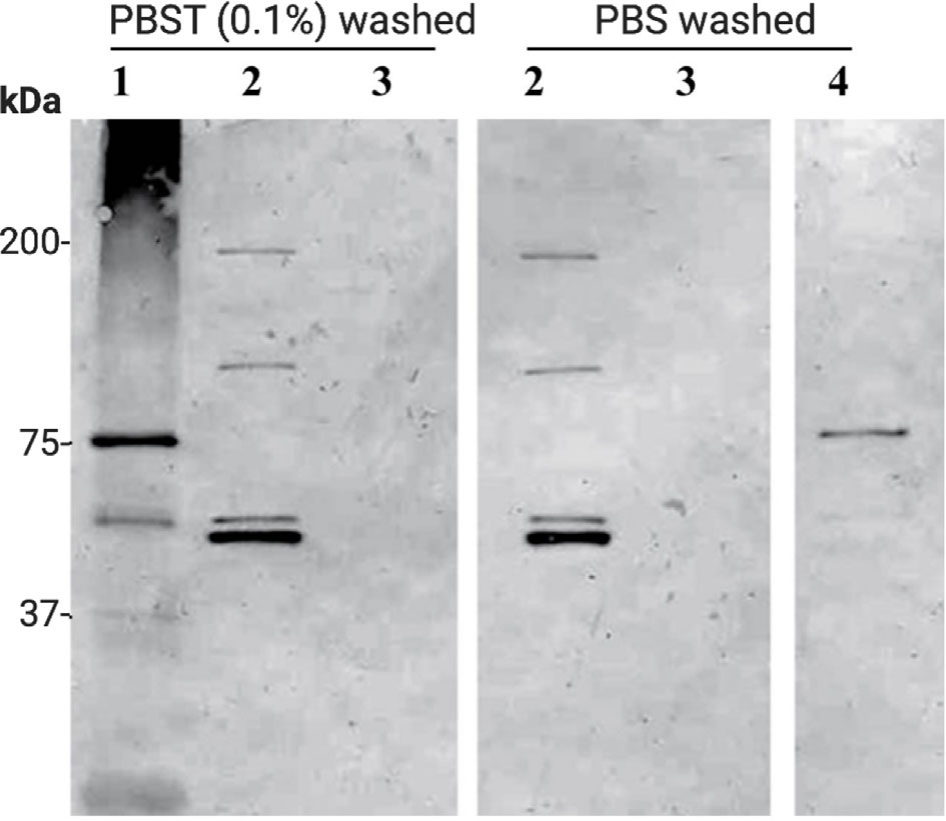
Affinity pull-down of *Plasmodium gallinaceum* chitinase complex from serum free ookinete culture supernatant. Chitin beads were added to serum free *P. gallinaceum* ookinete culture supernatants to affinity pull down the chitinase and chitinase-associated protein complex. The protein-bound beads were washed three times with PBS containing (0.1% Tween 20) (PBST) or PBS (2) to remove non-specific proteins. For control, chitin beads were incubated in M199 media alone (3), r*PgCHT1* spiked media (4), and *E. coli* lysate culture supernatant expressing soluble r*PgCHT1* (1) and processed as above. Then SDS sample buffer was added to the beads, boiled, and subjected to 4%–12% Tris-Glycine gradient SDS-PAGE. The protein was transferred to a membrane and probed with mouse anti-chitinase active site peptide (B993) antibodies. Lane 2 shows that the chitin beads successfully pulled down the chitinases and interacting high molecular chitinase and protein complex band. Washing with PBST did not detach the complex from the chitin beads. The duplex band (lane 2, around 50 kDa, bottom panel) shows the pro-enzyme and mature forms of *PgCHT1*; and two other higher immuno-reactive bands (100 kDa and just below 200 kDa) are homo or hetero-multimeric forms of a complex. The soluble *E. coli*-produced rPgCHT1 (1) was specifically pulled down by the chitin beads and did not show a higher molecular weight band.

Western immunoblot analysis performed on chitin bead pull-down of *P. gallinaceum* ookinete conditioned medium showed two chitinases recognized by the mouse anti-chitinase active site peptide antibody (**Figure 4**) (anti-chitinase active site-derived peptide antibody, B993), as shown (Vinetz and Kaslow, 1998; Vinetz et al., 2000). Bands were present at >200 and >75 kDa, and a duplex band at approximately 50 kDa. A silver-stained SDS-PAGE gel for the same sample showed an enriched duplex band at the corresponding 50 kDa immuno-reactive band (**Supplementary Figure 3**). An additional band at 37 kDa enriched by the chitin bead pull-down was not recognized by the anti-chitinase active site antibodies [B993, (Vinetz and Kaslow, 1998; Vinetz et al., 2000)] and is predicted to be *PgCHT2* (**Supplementary Figure 3**), consistent with our hypothesis that the high molecular weight chitinase complex represents specific protein-protein interactions, which are reduction sensitive (Langer and Vinetz, 2001), as previously observed. The duplex bands are consistent with previous data that indicate the presence of *PgCHT1* with a pro-domain and mature form of the enzyme (Vinetz et al., 2000). The higher molecular bands are consistent with the gel filtration chromatography data (see above) that indicate chitinase to be part of a high molecular weight complex. The high molecular bands are consistent with the gel filtration chromatography results and indicate that chitinase is a major protein in the hetero-multimeric complex secreted from ookinetes.

**Figure 5:**
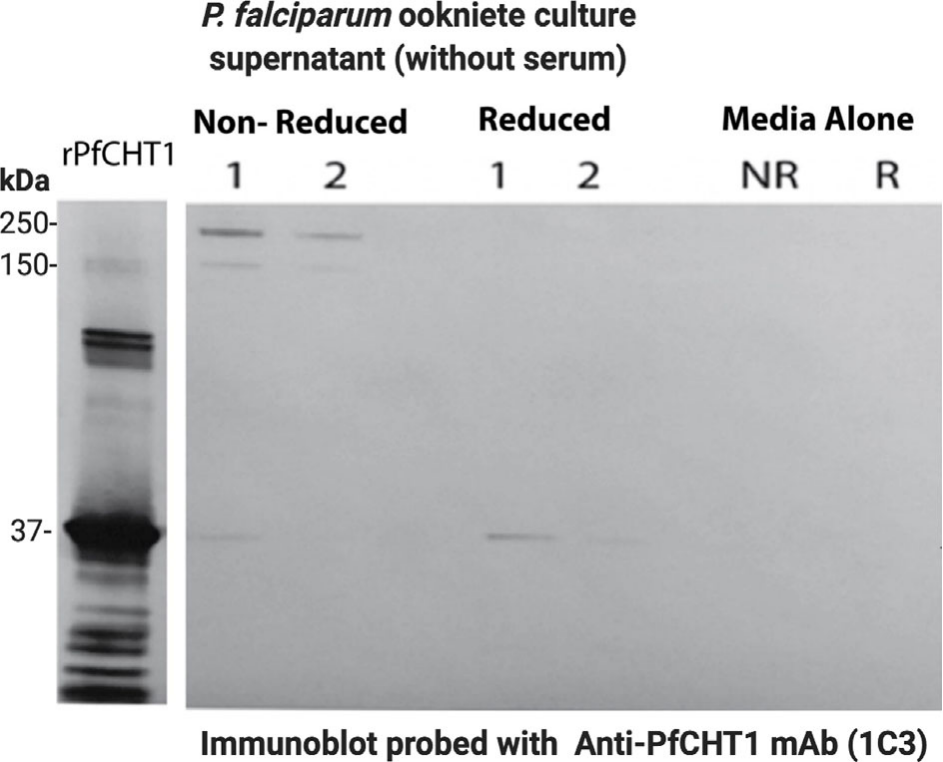
Western immunoblot of affinity pull-down of *Plasmodium falciparum* chitinase complex from ookinete culture supernatants. Washed chitin beads were added to the serum-free *P. falciparum* ookinete culture supernatants in replicate preparations (1, 2) to affinity pull down the chitinase and chitinase-associated protein complex. The protein bound beads were washed three times, SDS sample buffer (non-reduced, NR and reduced or R) was added to the beads, boiled, subjected to SDS-PAGE, and Western immunoblot analysis using anti-chitinase mAb (1C3). The samples were run under reduced (Red or R) and non-reduced conditions (Non-Red or NR) along with chitin beads incubated in ookinete media alone (NR and R) as controls. r*PfCHT1* protein was used as a positive control that is known to be recognized by mAb 1C3. Both serum-free media showed a high molecular chitinase complex in non-reduced samples and the same sample showed a smaller band of 37 kDa. The results were similar to observations of serum-containing ookinete medium (**Supplementary Figure 3**).

With *P. falciparum*, the chitin affinity pull-down complex from both serum-free (**Figure 5**) and serum-containing (**Supplementary Figure 4**) ookinete culture supernatants were resolved using SDS-PAGE under reduced and non-reduced conditions, followed by western immunoblot probed with the anti-chitinase monoclonal antibody, 1C3. Reduction with DTT eliminated the high molecular mass *P. falciparum* chitinase-containing complex, indicating dependency on disulfide linkages for forming a HMW complex. However, the monomeric form of *PfCHT1* (37 kDa) was also observed under non-reduced conditions, suggesting that the monomeric/free form of *PfCHT1* is also secreted by mature ookinetes. The mass spectrometry analyses of the chitinase complex formation were similar with and without human serum in the ookinete transformation media, indicating that the high molecular weight complex is of parasite origin.

### Mass Spectrometry Identified Key Micronemal Proteins in the Short Chitinase-Containing Chitin Affinity Pull-Downs of *Plasmodium gallinaceum* and *Plasmodium falciparum* Ookinete-Conditioned Media

Mass spectrometry analysis was carried out directly on the affinity pull-down chitin beads to identify the proteins present in the high molecular weight chitinase complex in both *P. gallinaceum* (**Table 2**) and *P. falciparum* (**Table 3**). *PgCHT1* and *PgCHT2* were identified (spectra counts of 32 and 53 respectively) along with equally enriched ookinete micronemal proteins known to play roles in mosquito midgut invasion (Dessens et al., 2003; Li et al., 2004; Ramakrishnan et al., 2011), including WARP (spectra count 36) and ookinete-surface enolase (Ghosh et al., 2011) (spectra count 30). A plasmepsin [an aspartic protease (Li et al., 2010)] was detected in the chitin bead pull-down material, but the spectra count was too low to conclude an association with the complex (**Supplementary Data File 2**). Taken together, these results show that *P. gallinaceum* chitinases are present in a complex along with key proteins that are essential for mosquito midgut invasion. The reduction sensitivity suggests that the integrity of the complex depends either on intermolecular disulfide bonds or intramolecular bonds within essential protein globular domains.

**Table 2:** List of proteins identified by mass spectrometry in the *P. gallinaceum* chitinase complex.

Locus* P. gallinaceum*(Mol. wt/length aa)	Protein name	Spectracount (seq count)	Known function in ookinete stages
PGAL8A_00473200 (42 kDa, 733 aa)	Chitinase, *PgCHT2* (short form chitinase)	53 (37)	Invasion
PGAL8A_00321200 (68 kDa, 587 aa)	Chitinase, *PgCHT1* (long form chitinase)	32 (18)	Invasion
PGAL8A_00077400/ (33 kDa, 292 aa)	von Willebrand factor A domain-related protein (WARP)*^a^*	38 (18)	Cell adhesion
PGAL8A_00392100/ (48 kDa, 446 aa)	Enolase	30 (16)	Interact with mosquito gut protein (EBP)
PGAL8A_00471000/ (301 kDa, 2257 aa)	G377	26 (18)	Not known
PGAL8A_00185600 (24 kDa, 207 aa)	Gamete antigen*^a^*	18 (8)	Not known
PGAL8A_00016000/ (328 KDa, 2847 aa)	P230*^a^* 6-Cys protein	16 (8)	Gamete-gamete interaction
PGAL8A_00344300 24 kDa, 214 aa	GTP binding Ran TC4	7 (5)	Unknown signal pathway

Mass-spectrometry identification of proteins that are associated with P. gallinaceum chitinase complex purified via affinity pull-down by chitin beads. Multidimensional Protein Identification Technology (MudPIT) was carried out directly on the beads that were incubated in the P. gallinaceum ookinete culture supernatant to affinity pull down the chitinase complex. Chitin beads were washed with PBS three times before subjected to MudPIT and the presence of chitinase complex confirmed by western immunoblot. MS/MS spectra verified against chicken (Gallus gallus) protein entries and a manually added list of common contaminants (e.g. human keratin and trypsin). The final list of peptides sequences was used for BLAST of the PlasmoDB databases (PlasmoDB.org) to identify the updated list of proteins. Only the spectra which had more than five spectra counts are presented here.

^a^These proteins were also seen in the gel slice representing the high molecular complex that was positive by western blot with anti-chitinase (1C3) mAb.

**Table 3:** Mass spectrometry analysis of the *Plasmodium falciparum* chitinase complex.

Locus *P. falciparum* orthologs (Mol. wt/length aa)	Protein name	Spectra count	Known function in ookinete stages
PF3D7_1252200 (42 kDa, 733 aa)	Chitinase, *PfCHT1*	17	Invasion
PF3D7_0801300 (32 kDa, 290 aa)	von Willebrand factor A domain-related protein (WARP)	12	Cell adhesion
PF3D7_1404300 (23 kDa, 202 aa)	Secreted ookinete adhesive protein (SOAP)	3	Interaction with mosquito gut protein (EBP)

Mass-spectrometry identification of proteins that are associated with P. falciparum chitinase complex purified by chitin bead affinity pull-down. Multidimensional Protein Identification Technology (MudPIT) was carried out directly on the beads that were incubated in the P. falciparum ookinete culture supernatant to affinity pull down the chitinase complex. Before subjected to MudPIT the chitin beads were washed with PBS three times and confirmed for the presence of chitinase complex by western-immunoblot. MS/MS spectra verified against protein entries manually added to a list of common contaminants (e.g. human keratin and trypsin) and normalized against zygote culture supernatant-chitin bead pull-down samples. The final list of peptides sequences was analyzed by BLAST against PlasmoDB (PlasmoDB.org) to identify the proteins. Out of 17 total identified proteins in the sample beads (affinity pull-down chitinase complex- chitin pull-down), only three proteins showed more than two peptides in normalized samples. In the zygote pull-down samples, chitinase, WARP, and SOAP showed low spectral counts 1, 0, and 0, respectively, indicating that they are present only in an ookinete-secreted complex.

For *P. falciparum* chitin bead pull-down samples *PfCHT1* was found as an abundant protein along with WARP, fructose-bisphosphate aldolase, gamete antigen 27/25, and SOAP. The mass spectrometry analysis of SDS-PAGE gel slices including the *PfCHT1* HMW complex and the 37 kDa *PfCHT1* (**Figure 5**, **Supplementary Data File 3**) further confirmed that WARP is a key, covalently-linked or otherwise reduction-sensitive PfCHT1 partner involved in the high molecular weight chitinase complex, and is also found in the chitin bead affinity pull-down mass spectrometry samples (**Supplementary Data File 4**).

### PgCHT1 Conserved Cysteines Involved in Chitin Binding *Via* Analysis of Site-Directed Mutagenesis and *rPfCHT1* Binds to Chitin Beads That Is Resistant to High Detergent Wash

**Figure 6:**
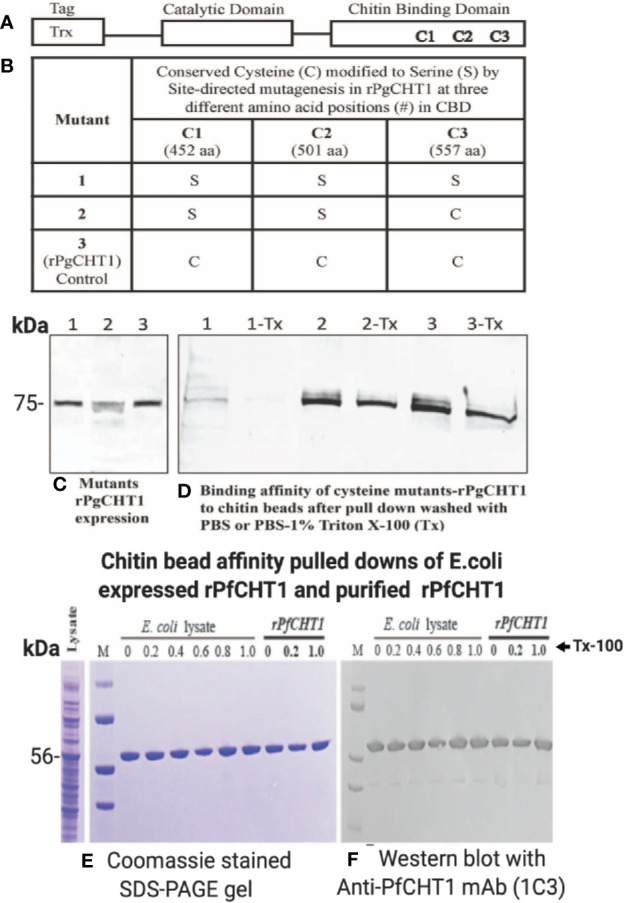
Site-directed mutagenesis of conserved cysteines located in the chitin binding domain of *P. gallinaceum* chitinase (*rPgCHT1*) and evaluation of chitin binding affinity of mutant *rPgCHT1* and *rPfCHT1*. Three conserved three cysteines **(A)** that were selected for mutation using the QuickChange Multi Site-Directed Mutagenesis Kit (Stratagene) with the known template (*PgCHT1*- PET32b plasmid DNA expression construct) containing a thioredoxin epitope tag (TRX-Tag). The changes of single base pairs were achieved within codons 452 aa (1,355 bp, TGC to TCC), 501 aa (1,502 bp, TGC to TCC) and 557 aa (1,670 bp, TGT to TCT) of the plasmid constructs to mutate cysteine **(C)** residues to serine (S) as shown in **(B)**. The *PfCHT1*- pET-32b expression plasmid was obtained from Gene Universal (Newark DE). The #1 mutant plasmid (in which all three C were modified), #2 plasmid (only two C modified), and wild type (3, *rPgCHT2*-PET32) and *rPfCHT1* were transformed into SHuffle T7 Express Competent *E. coli* cells for protein expression. The *rPgCHT1* lysates were incubated with chitin beads, washed three times either with either PBS (1, 2, 3) or PBS with 1% Triton X-100 (1-Tx, 2-Tx, and 3-Tx) to remove non-specific or loosely bound chitinase, and evaluated for binding affinity of the mutant rPgCHT1. Similarly, primary *E. coli* lysate containing rPfCHT1 in soluble form Ni-NTA purified rPfCHT1 was incubated with chitin beads and washed with PBS and PBST (0.2%–1% Triton-X 100). The bound chitin beads were mixed with sample buffer and subjected to SDS-PAGE followed by Western immunoblot analysis with anti-thioredoxin monoclonal antibody (Abcam) to detect rPgCHT1, and 1C3 antibody to detect rPfCHT1 **(E, F)**. Panel **(C)** shows the expression of rPgCHT1 (75 kDa) protein in all clones. The binding affinity to chitin beads of mutant #58 (all three cysteines modified) was lower and largely eliminated with the Triton-X 100 wash **(D)**, indicating that the chitin binding domain is essential for the interaction of the long form chitinase (*PgCHT1*) to chitin. Panels **(E, F)** show the strong binding of rPfCHT1 to chitin beads in both **(E)** *coli* culture lysates and purified rPfCHT1. In **(E, F)**, chitin beads were washed with PBS with different concentrations of Triton X 100 (0%–1%). rPfCHT1 strongly binds to chitin beads despite lacking a chitin binding domain.

The long form chitinase (*PgCHT1*) gene encodes a protein possessing a putative chitin binding domain (CBD) (Vinetz et al., 2000), in contrast to the short form chitinases (PgCHT2 and PfCHT1). Earlier experiments showed that both the long and short form chitinases binds to chitin. Here we examined the putative role of the three conserved cysteines in the PgCHT1 CBD by site directed mutagenesis to individually replace each cysteine to serine. Mutated rPgCHT1 proteins were expressed in E. coli and their binding to chitin beads compared to wild type protein (**Figure 6**). Mutated rPgCHT1 protein with three conserved cysteines replaced with serine in the CBD (clone 1) bound to the chitin beads was removed by the detergent wash (PBS with 1% Triton-X 100), whereas wild type bound protein resisted the detergent wash. This result demonstrates that a chitin binding domain is involved in PgCHT1 high-affinity interactions with chitin.

To assess the specificity of the chitin bead pull-down assay, rPfCHT1 (**Figures 6D, E**) was pulled down purely from gross rPfCHT1 *E. coli* lysate and PBS spiked with purified rPfCHT1; specifically, it was observed as a single band without non-specific contamination in Coomassie stained SDS-PAGE gels. PfCHT1 lacking a putative chitin binding domain showed high affinity binding for the chitin beads and resisted washing with high concentrations of detergent (1% Triton X-100 in PBS). It will be of interest to delineate which regions of PfCHT1 specifically bind to the solid phase chitin substrate.

### Generation and Characterization of a *Plasmodium berghei* Transgenic Gene Insertion/Marker Out Parasite Line

**Figure 7:**
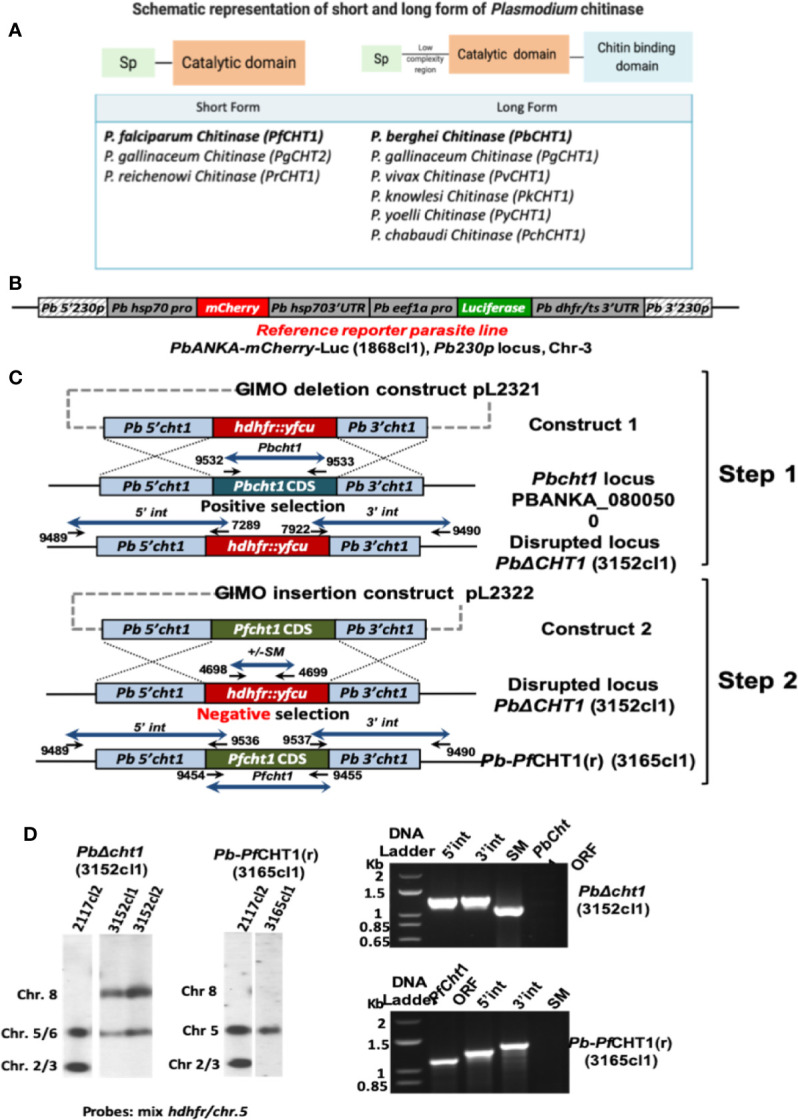
Generation of the chimeric *P. berghei* parasite line, *Pb-PfCHT1*(r), expressing the short form of *P. falciparum* chitinase (*PfCHT1*). **(A)** Schematic representation of the short and long forms of chitinase found in different *Plasmodium* species. The short form chitinases lack the proenzyme and chitin-binding domains, compared with the long forms of chitinases which have proenzyme and chitin-binding domain in addition to substrate-binding and catalytic domains. **(B)** Schematic representation of the *Pb230p* locus of the reference reporter *P. berghei* ANKA parasite 1868cl1 which was used to generate the chimeric *Pb-PfCHT1*(r) parasite line (see C). This reporter line is selectable marker (SM)-free and expresses mCherry protein under the strong constitutive *Pbhsp70* promoter and firefly luciferase (LUC-IAV) under the constitutive *Pbeef1a* promoter. The reporter-cassette is integrated into the neutral *230p* locus in chromosome 3. **(C)** Schematic representation of the generation of the chimeric line *Pb-PfCHT1*(r) (line 3152cl1). First step: the GIMO deletion-construct (construct 1; pL2321) was used to replace the *Pbcht1* coding sequence (CDS) with the positive/negative selectable marker (SM; h*dhfr::yfcu*) cassette, resulting in the generation of the *PbΔcht1* (line 3152cl1) after positive selection with pyrimethamine. Construct 1 targets the *Pbcht1* gene by double cross-over homologous recombination. After genotyping and confirmation of correct construct integration, this line was cloned by limiting dilution. Second step: The GIMO insertion construct (construct 2) was used to replace the SM in the *PbΔCHT1* GIMO line with the *Pfcht1* CDS, resulting in the generation of line *Pb-PfCHT1*(r) (line 3165cl1) after negative (5-FC) selection. Construct 2 integrates by double cross-over homologous recombination using the same targeting regions employed in construct 1, resulting in the introduction of the *Pfcht1* CDS under the control of *Pbcht1* regulatory sequences. Black arrows: locations and primer numbers used for diagnostic PCR. **(D)** Genotype analysis of *PbΔcht1* and *Pb-PfCHT1*(r) parasites by Southern analysis of chromosomes (chr) separated by pulsed-field gel electrophoresis (PFGE) (left) and diagnostic PCR analysis (right). Hybridisation of PFG-separated chr of *PbΔcht1* with a mixture of *hdhfr* and a probe specific for chr 5 confirms integration of construct 1 into the *Pbcht1* gene on chr 8. The correct integration of construct 2 in *Pb-PfCHT1*(r) was confirmed by showing the removal of the *hdhfr::yfcu* selectable marker (SM) cassette by hybridisation of chr with the *hdhfr* and chr 5 probe. As an additional control (ctrl), parasite line 2117cl1 was used with the *hdhfr::yfcu* SM integrated into chr 3. Diagnostic PCR analysis confirms the deletion of *Pbcht1* in *PbΔcht1* and the correct integration of the *Pfcht1* expression cassette in *Pb-PfCHT1*(r). Correct integration is shown by the absence of the *hdhfr::yfcu* SM and the *Pbcht1* CDS, the presence of the *Pfcht1* CDS, and correct integration of the construct into the genome at both the 5’ and 3’ regions (5’int and 3’int; see B for primer numbers and locations). Primer sequences and expected PCR product sizes are shown in **Supplementary Table 1**.

A chimeric *P. berghei* parasite line was produced in which the coding sequence (CDS) of the *P. berghei* chitinase gene (*PbCHT1*; PBANKA_0800500) was replaced with the CDS of *P. falciparum* chitinase (*PfCHT1*; PF3D7_1252200), amplified from genomic DNA of *P. falciparum* NF54 (**Figure 1**). This chimeric line, *Pb-PfCHT1*(r), constitutively expressed mCherry and luciferase as reporter proteins throughout the life cycle. We confirmed the correct replacement of the *PbCHT1* CDS by the *PfCHT1* CDS in the chimeric line by Southern analysis of chromosomes separated by pulsed-field gel electrophoresis and by diagnostic PCR on genomic DNA (**Figure 7**). These parasites were then characterized as described below.

**Figure 8:**
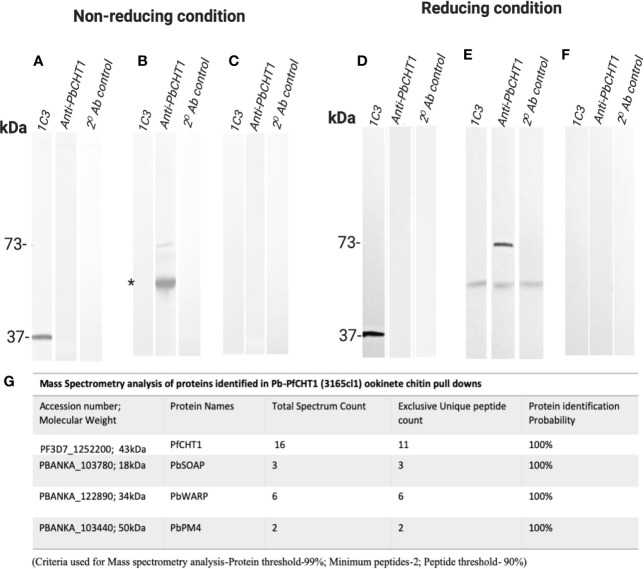
Western Immunoblotting of chitin affinity pull-downs of *Pb-PfCHT1 (r)*, *PbCHT1 (WT)*, and *PbCHT1-KO* from *in vitro* ookinete culture supernatants: Figures show under non-reducing conditions **(A–C)** and under reducing conditions **(D–F)**. To pull down the chitinase (mainly PfCHT1)-associated microneme protein partners from the ookinete culture supernatants, washed chitin beads were added and incubated at 4°C for 4 h on a rotary shaker. Chitin beads were washed three times with PBST (0.5% Tween 20) followed by three washes with PBS (pH 7.4) and suspended in SDS sample buffer (+/− 2-mercaptoethanol). SDS-PAGE and western immunoblots were performed and probed with anti-PfCHT1 monoclonal antibody (1C3) and anti-PbCHT1 polyclonal antibody. A secondary antibody control was run with each blot under both conditions. **(A, D)** Pb-PfCHT1, under non-reducing and reducing conditions. Reactivity of native PfCHT1 (37 kDa) was seen exclusively with 1C3 monoclonal antibody and not with polyclonal anti-PbCHT1 under both conditions. No specific HMW band was detected with 1C3. **(B, E)** PbCHT1, under non-reducing and reducing conditions. Presence of native PbCHT1 (73 kDa) was observed specifically with polyclonal anti-PbCHT1 antibody and not with 1C3 in both NR and R conditions. Some non-specific reactivity (*) was observed when probed with 1C3 and anti-PbCHT1 and also in a secondary antibody control blot. **(E, F)** PbCHT1-KO, under non-reducing and reducing conditions. Native PbCHT1 was not observed either with 1C3 or polyclonal anti-PbCHT1 antibody. Secondary antibody controls were run with each blot, where no non-specific reactivity was observed. **(G)** Table represents the mass spectrometry analysis of proteins identified in Pb-PfCHT1 (3165cl1) ookinete culture supernatants chitin pull-downs. The presence of microneme proteins secreted ookinete adhesive protein (SOAP), von Willebrand factor A domain-related protein (WARP), and PM4 were observed. The data shows the absence of the high molecular weight (HMW) complex in the Pb-PfCHT1 parasite lines despite the presence of the partner proteins (*P. berghei* specific). This might suggest the importance of the species specific chitinase binding partner proteins to be present in order to form the HMW complex in a *P. berghei* mouse model.

*P. berghei* ookinetes were equally well produced *in vitro* by all parasite lines. *P. berghei in vitro* ookinete culture supernatants from all three parasite lines (*Pb-PfCHT1(r)*, *PbCHT1 (WT)*, and *PbΔCHT1*) were used for the evaluation of the HMW multimeric complex using the chitin bead affinity pull-down assay. Washed chitin beads were subjected to SDS-PAGE and western immunoblots under both non-reducing and reducing conditions and probed with 1C3 and anti-*PbCHT1*-polyclonal antibody (**Figures 8A–F**). The successful expression of the native, monomeric form of *PfCHT1* (37 kDa) and *PbCHT1* (73 kDa) proteins were observed in the ookinete culture supernatants from the respective *Pb-PfCHT1(r)* and *PbCHT1 (WT)* parasite lines with the complete absence of *PbCHT1* in the *PbΔCHT1* parasite cell line ookinete culture supernatant. However, the chitin affinity pull-down was able to pull down monomeric *PfCHT1* (37 kDa) efficiently. The presence of a high molecular mass protein complex in the *Pb-PfCHT1*(r) (**Figure 8A**) *in vitro* ookinete culture supernatants was not observed when probed with 1C3 under non-reducing conditions.

**Figure 9:**
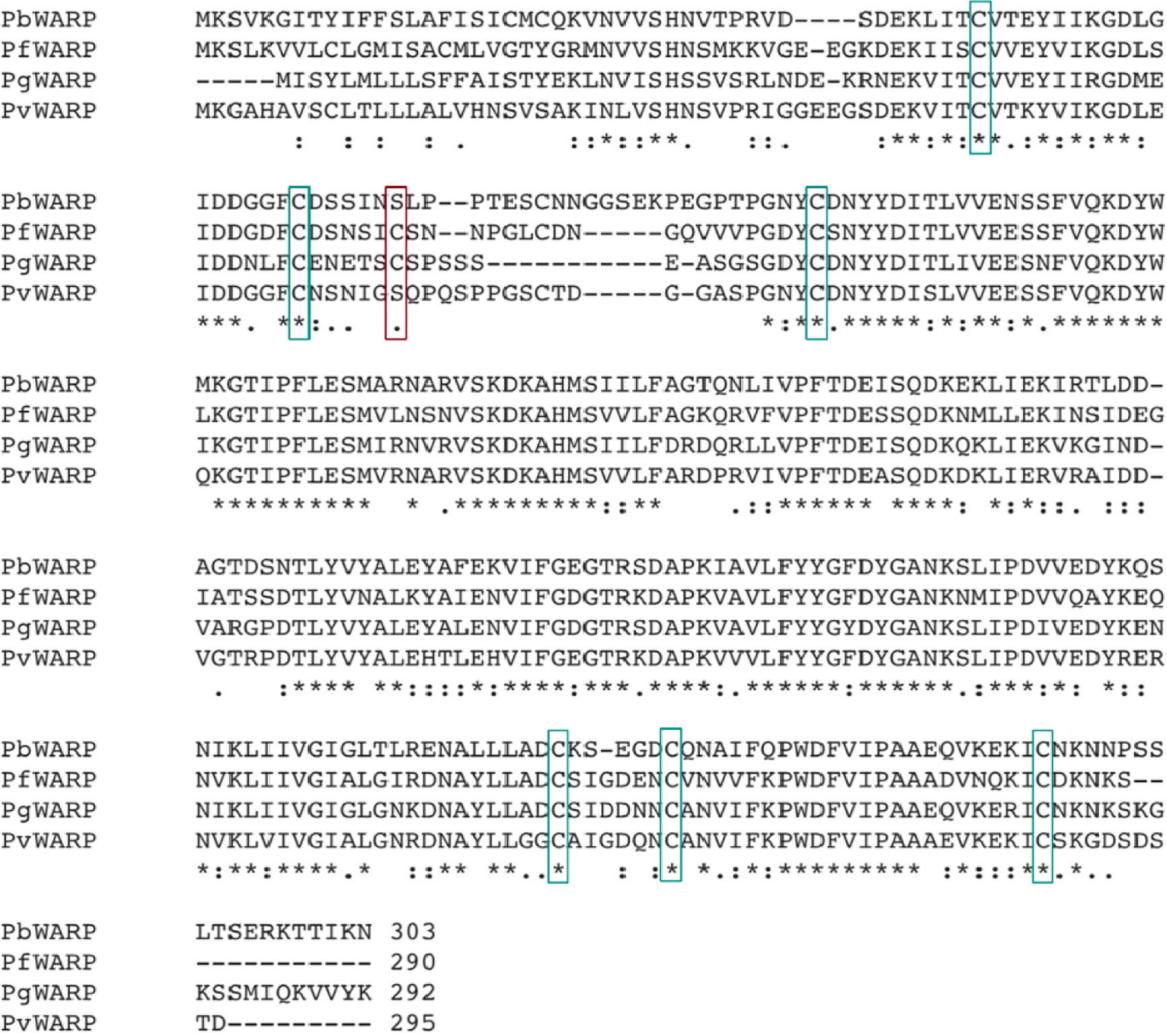
Identification of a putative cysteine (C) residue involved in the formation of the multimeric high molecular chitinase complex of *Plasmodium gallinaceum* and *Plasmodium falciparum*. Multiple sequence alignment (MSA) of full-length von Willebrand factor A domain-related protein (WARP) proteins of *Plasmodium berghei*, *P. falciparum*, *P. gallinaceum* and *P. vivax*. Fully conserved amino acid residues are shown by an asterisk (*), amino acids showing strongly similar properties are represented by a colon (:) and weakly conserved by a period (.). The *Plasmodium* species with a short form chitinase (*P. falciparum, PfCHT1*; *P. gallinaceum*, *PgCHT2*) have one conserved cysteine residue in the N-terminal structure of the WARP protein compared to all long form chitinases (*PbCHT1* and *PvCHT1*) which instead have a serine (shown in red rectangles). The conservation of the cysteine residue in *P. falciparum* and *P. gallinaceum* suggests a species-specific WARP - short chitinase covalent interaction responsible for the formation of the reduction-sensitive multimeric high molecular weight chitinase complex. The lack of a HMW complex formation in transgenic *P. berghei* heterologously-expressing *PfCHT1* supports this hypothesis. The other cysteine residues are highly conserved in the other *Plasmodium* WARP proteins depicted.

Mass spectrometry analysis was carried out on the chitin affinity pulled down proteins from the *Pb-PfCHT1(r)* ookinete culture supernatant (**Figure 8G**). A high spectrum count of 16 was found for the native *PfCHT1* followed by low spectra counts for other microneme proteins that play important roles in mosquito midgut invasion (Dessens et al., 2003; Li et al., 2004). Other than the *P. berghei* von Willebrand Factor A domain-related protein (*PbWARP*; spectral count 6) and *P. berghei* secreted ookinete adhesive protein (*PbSOAP*; spectral count 3) proteins, an aspartic protease *P. berghei*. Plasmepsin IV (*PbPM4*) with low spectra count (**Figure 8G**) was also detected *via* mass spectrometry (**Supplementary Data File 5**). The mass spectrometry proteomics data have been deposited to the ProteomeXchange Consortium *via* the PRIDE partner repository with the dataset identifier PXD021970 (Perez-Riverol et al., 2019). To explore a possible reason for the absence of the HMW hetero-multimeric complex in the *Pb-PfCHT1(r)* ookinete culture supernatant, we examined differences in the primary structure of the chitinase binding protein partner WARP. The *Plasmodium* species with short form of the chitinases (*PfCHT1* and *PgCHT2*) have a conserved cysteine residue in the N-terminal region of the WARP protein, in contrast to a serine residue at this position in species possessing the long form of the chitinases (*PbCHT1* and *PvCHT1*). The conserved cysteine is possibly unpaired intramolecularly, and instead might participate in cross-linkages and stabilize partner proteins secreted as a HMW complex in *in vitro P. falciparum* ookinete culture supernatants (**Figure 9**).

## Discussion

Here we demonstrate that *Plasmodium falciparum* and *P. gallinaceum* ookinete secrete a short-form chitinase as a covalently-linked, reduction-sensitive, high molecular weight hetero-multimeric protein complex with highest approximate mass of 1,300 kDa. Proteomic analysis of material pulled down using chitin beads shows that this complex contains both reduction-sensitive, i.e. cysteine-disulfide bonded components (short chitinase-WARP) and SDS-sensitive components (non-covalently associated proteins such as SOAP and enolase). This complex potentially enables the *Plasmodium* ookinete to invade the mosquito midgut, and characterization of this pathway might reveal targets for malaria transmission blocking strategies. The complex appears to contain WARP, consistent with previous data (Li et al., 2004), and possibly other ookinete-expressed proteins, as suggested by mass spectrometry analysis of proteins pulled down using a chitin affinity assay as well as a gel slice that represents the anti-chitinase (1C3) positive HMW chitinase complex.

To determine whether the presence of this secreted, chitinase-containing invasion complex is generalizable to other *Plasmodium* species, we studied *P. gallinaceum*, an avian-infecting *Plasmodium* species which is a well-established model for studying ookinete biology, and *P. falciparum*, the major human pathogen for which ookinetes have only relatively recently been able to be produced *in vitro* (Dinglasan et al., 2007a; Dinglasan et al., 2007b; Bounkeua et al., 2010; Ghosh et al., 2010). There are two types of active chitinases in *P. gallinaceum* (PgCHT1 and PgCHT2, long and short form respectively) and only one short form of active chitinase present in *P. falciparum* (PfCHT1). *P. vivax* and other human-infecting malaria parasites encode only the long form chitinase (PvCHT1), homologous to PgCHT1 (Tsuboi et al., 2003). Published data showed the presence of a high molecular weight chitinase in *P. gallinaceum*, by anion-exchange chromatography of ookinete supernatants (Vinetz et al., 2000) and by western immunoblot using an anti-chitinase monoclonal antibody (1C3, a monoclonal developed against the active site of PfCHT1 chitinase that also recognizes an identical epitope in the short form of chitinase in *P. gallinaceum*, PgCHT2) (Langer et al., 2002a). However, we were unable to use antibodies to pull down the chitinase complex, possible due to steric hindrance or low affinity binding of antibodies to native enzymes or sequestered epitopes within the high molecular weight complex. We were able to overcome this technical problem by using chitin beads, which enabled us to identify the components of the high molecular mass chitinase-containing complexes in two phylogenetically distant *Plasmodium* species. The chitin bead pull-down method was successfully used to purify native parasite secreted chitinases as well as the recombinant chitinases rPfCHT1 and rPgCHT1. The data shows that rPfCHT1 (**Figures 6D, E**) was purified neatly from *E. coli* lysate without non-specific bands in Coomassie stained gels and resisted a high detergent wash. Unpublished data (Kaur and Vinetz, 2020) show that modification of conserved cysteines in the rPfCHT1 almost eliminates binding to chitin beads, versus its high affinity binding in native forms.

Ookinete-secreted chitinases are essential for the malaria parasite to establish mosquito infection, and hence continuation of the *Plasmodium* life cycle and malaria transmission (Huber et al., 1991; Sieber et al., 1991; Shahabuddin et al., 1993; Shahabuddin and Vinetz, 1999; Vinetz et al., 1999; Vinetz et al., 2000; Langer and Vinetz, 2001; Li et al., 2005). The ookinete-secreted native high molecular weight native chitinase complex, identified by size exclusion chromatography for both *P. gallinaceum* and *P. falciparum*, is reduction-sensitive suggesting that this complex is either produced in the ookinete secretory pathway as a cysteine-dependent hetero-multimer or that disulfide bond-stabilized protein globular domains are involved in the complex formation. Mass spectrometry analysis demonstrated that interacting partners of chitinase are other known ookinete proteins, including WARP, enolase and SOAP, which are known to play a role in midgut invasion (Dessens et al., 2003; Abraham et al., 2004; Li et al., 2004). In *P. gallinaceum*, both *PgCHT1* and *PgCHT2* are part of the complex, along with the micronemal proteins WARP and SOAP that were also identified in *P. falciparum*.

*P. falciparum* ookinetes were produced *in vitro* using serum-containing medium [16, 17] and these methods were not suitable for our studies due to chitinase activity of the serum. Therefore, we successfully produced ookinetes of *P. falciparum in vitro* using serum free conditional medium and the supernatant was used to affinity pull-down the chitinase using chitin beads. A *PfCHT1* high molecular mass complex was pulled down from ookinete culture supernatant by chitin beads, recognized by anti-PfCHT1 mAb (1C3), and confirmed by mass spectrometry analysis. The mass spectrometry analysis of a gel band corresponding to the high molecular weight *P. falciparum* identified WARP protein, similar to its presence in the *P. gallinaceum*-secreted chitinase complex.

To our knowledge this report is the first to demonstrate the presence of P. falciparum ookinete-produced native chitinase, which necessitates further explorations of its biological functions. While we recognize the limitation of the chitin bead affinity pull-down of native chitinase for mass spectrometry analysis, which might have non-specifically pulled down ‘sticky’ micronemal proteins, our size exclusion chromatography analysis of *P. falciparum* and *P. gallinaceum* ookinete-secreted chitinases showed both reduction-sensitive (WARP) and SDS-sensitive components (SOAP, enolase) of the high molecular complex. The high degree of purity of chitin bead pulldown of rPfCHT1 from recombinant *E. coli* lysate (**Figure 6**) supports the specificity of this method for detecting chitin-binding activity along with proteins that bind to the chitinase and therefore are indirectly bound by chitin beads. Therefore, mass spectrometry analysis of an anti-chitinase immunoreactive gel slice representing the HMW, and comparing with mass spectrometry analysis of chitin pull-down samples confirms that WARP and other proteins are consistently present in the chitinase complex of both species.

Previous experimental work was not able to directly observe the *P. falciparum* ookinete-produced chitinase because of technical limitations, which were overcome in the present study. Moreover, while the *P. gallinaceum* ookinete-secreted long form of the chitinase has a predicted chitin-binding domain, *P. falciparum* chitinase, *PfCHT1*, does not (Vinetz et al., 1999). Mutation of three conserved cysteines within in the putative chitin-binding domain of *PgCHT1* using site directed mutagenesis showed, for the first time, that this domain is involved in chitin binding. Hence a key conclusion of the present work is that two *Plasmodium* ookinete-secreted chitinases with unrelated primary structures bind to chitin, as demonstrated by mass spectrometry analysis of a chitin bead pull-down assay as well as by size exclusion chromatography.

To further characterize the HMW complex using a genetic approach, the *P. berghei* model was developed by using a novel GIMO method to successfully replace the *PbCHT1* (long form) with *PfCHT1* (short form). The *in vitro* ookinetes culture supernatants revealed the absence of a reduction sensitive HMW chitinase-containing complex, confirming the importance of the species-specific binding protein partners in the complex formation. However, mass spectrometry analysis performed on the *Pb-PfCHT1(r)* pull-downs confirms the presence of other important micronemal proteins including PbWARP and PbSOAP that play a role in mosquito midgut invasion (Hirosawa et al., 1993; Dessens et al., 2003).The inability of heterologously-expressed PfCHT1 to form the high molecular weight complex in the *P. berghei* system could be attributed to a number of possibilities, one of which might be the absence of a suitable WARP protein with the necessary cysteine and other structural features that would enable covalent linkage to PfCHT1 and other micronemal proteins to form the short chitinase-containing high molecular weight complex (Li et al., 2004). This possibility is of fundamental interest and is the focus of ongoing experiments.

*Plasmodium* actively invades host cells without depending on host uptake pathways (Paing and Tolia, 2014), and parasites are known to have evolved diverse methods to form high avidity complexes for invasion (Wright and Rayner, 2014). There is growing evidence that multimeric assemblies of parasitic ligands and host surface molecules strengthen the host-parasite interactions necessary for invasion (Paing and Tolia, 2014). Sporozoites invade hepatocytes and the invasion complex of CSP and TRAP mediate the invasion process (Wengelnik et al., 1999). Merozoites invade red blood cells facilitated through a series of merozoite surface proteins (MSPs), including MSP complexes (Lin et al., 2016) and the AMA1-RON complex. It was recently shown that vaccination with the AMA1-RON2L protein complex produced protective antibodies providing a novel path for next-generation vaccine candidates against malaria (Srinivasan et al., 2014). Because the only known secretory organelles of ookinetes are micronemes, the microneme-secreted chitinase and its high molecular, chitinase-containing complex are a useful model system for understanding protein secretion mechanisms in *Plasmodium.* Given the recent successes in using the CRISPR/Cas9 system to modify genes in *Plasmodium* (Bansal et al., 2017; Knuepfer et al., 2017; Singer and Frischknecht, 2017; Vanaerschot et al., 2017; Zhang et al., 2017), the present work reveals future experiments to identify the mechanisms involved in protein secretion and secretory organelle formation, particularly in exploring the roles of the invariant cysteines in chitinase complex formation in the ookinete.

The mosquito midgut stages of the parasite are a critical bottleneck in the life cycle of *Plasmodium* (Bennink et al., 2016; Sinden, 2017b). The protein complexes formed at the mosquito malaria parasite stages are largely undetermined compared to the asexual stage protein complexes. Antibodies against zygote and ookinete surface proteins effectively block parasite development within the mosquito midgut (Patra et al., 2015); and these antigens are candidates of transmission blocking vaccine development (Dinglasan and Jacobs-Lorena, 2008; Atkinson et al., 2015; Delrieu et al., 2015; Patra et al., 2015; Brickley et al., 2016; Theisen et al., 2017). The protein complexes that are involved during ookinete invasion of the mosquito midgut are not understood either in terms of structure or precise function. The identification and functional assessment of these protein comprising the high molecular weight complex is important to understand their role played in mosquito midgut invasion. Therefore, novel interventions to interfere with high molecular weight complex formation in *Plasmodium* may arise from understanding the cell biology and biochemistry of novel malaria parasite development within the vector mosquito.

## Data Availability Statement

The mass spectrometry proteomics data have been deposited to the ProteomeXchange Consortium via the PRIDE [1] partner repository with the dataset identifier PXD021970; ProteomeXchange: PXD021911; and MassIVE: MSV000086264.

## Ethics Statements

The animal study was reviewed and approved by Yale University, Leiden University Medical Center, University of California San Diego.

## Author Contributions

KP, HK, SK designed and carried out experimental work and drafted the manuscript. JG carried out the chitin bead pull-down assays. JW and DG carried out the mass spectrometry of *P. falciparum* chitin pull-down proteins and analysis. HP and JY were responsible for the mass spectrometry of *P. gallinaceum* proteins, and CJ planned the chimeric *P. berghei* experiments and supervised the work. JV designed the experiments, originated and supervised the project, and finalized the manuscript. All authors contributed to the article and approved the submitted version.

## Funding

This work was supported by grants from the United States Public Health Service, National Institute of Health grants U19AI089681 (JV), AI45999 (JV), and P41 GM103533 (JY). The funders had no role in deciding to publish this work. JW was supported by the UCSD Graduate Training Program in Cellular and Molecular Pharmacology through the National Institute of General Medical Sciences (T32 GM007752) and the UCSD Training Program in Rheumatic Diseases Research through the National Institute of Arthritis and Musculoskeletal and Skin Diseases (T32 AR064194).

## Conflict of Interest

The authors declare that the research was conducted in the absence of any commercial or financial relationships that could be construed as a potential conflict of interest.

## Supplementary Material

Supplementary Figure 1 Chitinase activity of human serum, ookinete media, and method to deplete chitinase activity from media.

Supplementary Figure 2 Size exclusion HPLC fractionation of *Plasmodium gallinaceum* ookinete culture supernatant and analysis of chitinase activity.

Supplementary Figure 3 Silver staining of chitin bead affinity pull-down of *Plasmodium gallinaceum* chitinase complex.

Supplementary Figure 4 Serum containing media used for *P. falciparum* ookinete culture to affinity pull-down the *P. falciparum* chitinase complex.

Supplementary Table 1 List of primers used for generating transgenic *P. berghei* chimeric parasite lines expressing *PfCHT1* and their product sizes.

Supplementary Data Sheet 1 Estimation of HMW complex size by using high resolution chromatography and gel filtration standards and Blue Dextran.

Supplementary Data Sheet 2 Mass spec data of *P. gallinaceum* chitinase complex chitin bead pull-down from the serum free samples ookinete culture supernatant.

Supplementary Data Sheet 3 Mass spec data of *P. falciparum* chitinase complex chitin bead pull-down from the serum free samples ookinete culture supernatant.

Supplementary Data Sheet 4 Mass spec data of 1C3 (anti-*PfCHT1* mAb) positive gel bands under reduced and non-reduced conditions.

Supplementary Data Sheet 5 Mass spec data of Pb-PfCH1 (r) chitin bead pull-down samples from the samples from ookinete culture supernatant.
