# Supplementary material for: A Hetero-Multimeric Chitinase-Containing Plasmodium falciparum and Plasmodium gallinaceum Ookinete-Secreted Protein Complex Involved in Mosquito Midgut Invasion
Source: Front Cell Infect Microbiol. 2021 Jan 8;10:615343. doi: 10.3389/fcimb.2020.615343 (PMC7821095; doi:10.3389/fcimb.2020.615343)
Supplement: Supplementary Figure 1 — Chitinase activity of human serum, ookinete media, and method to deplete chitinase activity from media. [file DataSheet_1.pdf]

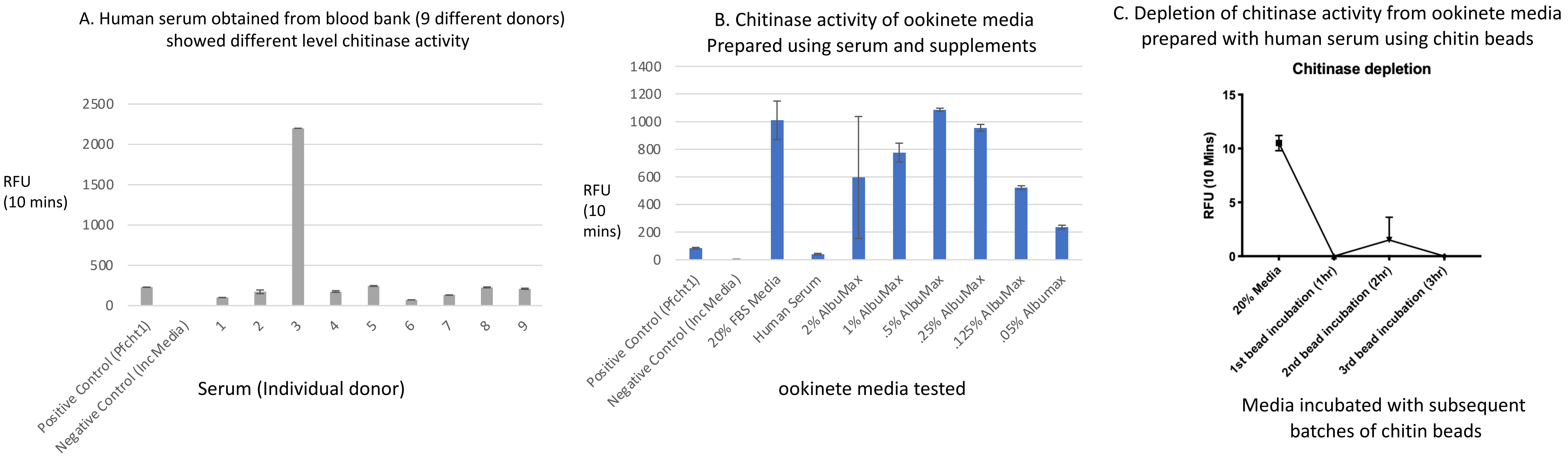

Supplementary Figure 1 : Chitinase activity of human serum from individual donors, ookinete media and method to deplete chitinase activity from media: Human serum obtained from National blood bank were evaluated for their chitinase activity (Panel A. 5 ul of serum sample diluted in 100 ul of PBS and checked for activity) and selected low chitinase activity samples for ookinete media preparation. Ookinete culture media prepared using human serum, Albumax and FBS and evaluated their chitinase activity (Panel B) and chitinase activity was was depleted (Panel C) by using incubation of media with chitin beads (3 batches, 50 mL of Media plus 0.5 mL of washed chitin beads) and chitinase activity was measured by standard methods.
