# Supplementary material for: A Hetero-Multimeric Chitinase-Containing Plasmodium falciparum and Plasmodium gallinaceum Ookinete-Secreted Protein Complex Involved in Mosquito Midgut Invasion
Source: Front Cell Infect Microbiol. 2021 Jan 8;10:615343. doi: 10.3389/fcimb.2020.615343 (PMC7821095; doi:10.3389/fcimb.2020.615343)
Supplement: Supplementary Figure 2 — Size exclusion HPLC fractionation of Plasmodium gallinaceum ookinete culture supernatant and analysis of chitinase activity. [file DataSheet_2.pdf]

HPLC fractionation of *Plasmodium gallinaceum* ookinete culture supernatant

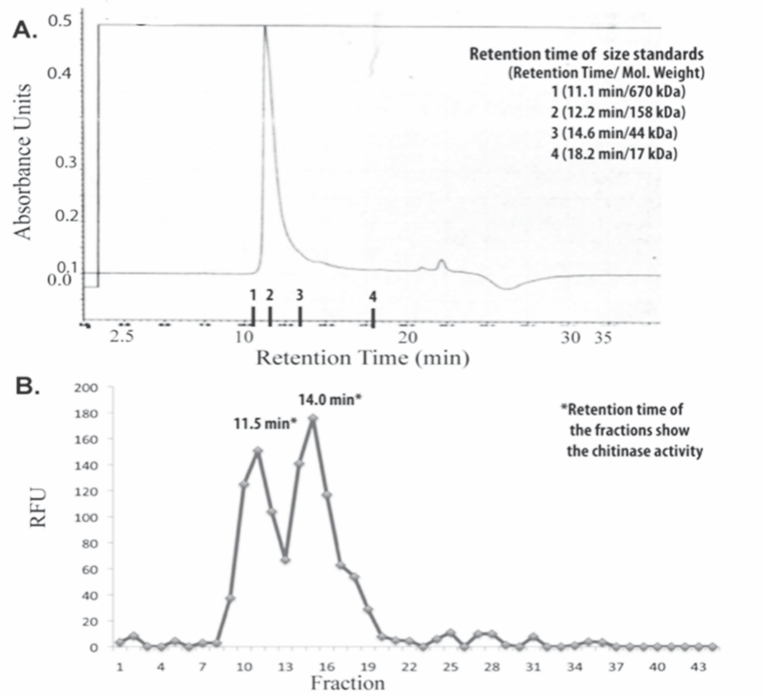

Chtinase activity assay in each eluted fractions (every minute) collected from HPCL Size Exclusion Column, the larger protein elutes early.

Supplementary Figure 2: Size exclusion HPLC fractionation of *Plasmodium gallinaceum* ookinete culture supernatant and analysis of chitinase activity. Approximately 50 mL of serum free ookinete culture supernatant was concentrated using Centricon (10 kDa cut off centrifugal filter devices, EMD Millipore, MT) to a final volume of 1 mL volume and loaded onto a TSKgel G2000SW column (Tosoh Biosciences LLC, King of Prussia, PA) using a Beckman Gold 500 HPLC instrument, Gel filtration standards (Bio-Rad) and autoclaved PBS (pH 7.4) were used for running the samples and fractions were collected. Approximately, 100 µl of sample from each fraction (Total 0.5 mL volume/one minute) were analyzed in duplicates for the presence of chitinase activity. We observed two distinct chitinase peak activity in *P. gallinaceum* ookinete secreted culture supernatant. To analyze further the sample were analyzed on a high resolution chromatography followedby immuno-detection and estimation of the chitinase complex in both *P. falciparum* and *P. gallinaceum* (Figure 5).
