# Supplementary material for: A Hetero-Multimeric Chitinase-Containing Plasmodium falciparum and Plasmodium gallinaceum Ookinete-Secreted Protein Complex Involved in Mosquito Midgut Invasion
Source: Front Cell Infect Microbiol. 2021 Jan 8;10:615343. doi: 10.3389/fcimb.2020.615343 (PMC7821095; doi:10.3389/fcimb.2020.615343)
Supplement: Supplementary Figure 3 — Silver staining of chitin bead affinity pull-down of Plasmodium gallinaceum chitinase complex. [file DataSheet_3.pdf]

Silver staining of chitin bead affinity pulldown of *Plasmodium gallinaceum* chitinase complex

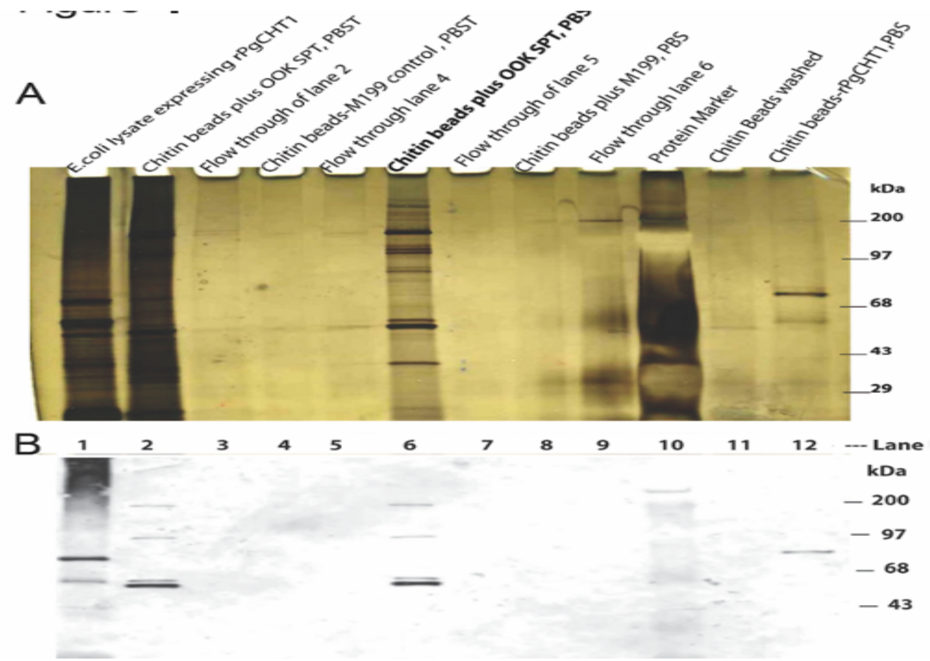

Supplementary Figure 3 : Silver staining of chitin bead affinity pulldown of *Plasmodium gallinaceum* chitinase complex. Chitin beads were added to serum-free *P. gallinaceum* ookinete culture supernatants as an affinity pulldown assay of the ookinete secretome to identify proteins associated with the chitinase complex. Protein bound beads were washed three times with PBS with 1% Tween 20 (PBST) or PBS (Lane 2 and 6 respectively). SDS sample buffer was directly added to beads, boiled, and analyzed by 4-12% Tris-Glycine gradient SDS-PAGE. As controls, samples were run along with chitin beads incubated in media alone (Lane 4 and 8), beads alone (lane 11), and incubated with rPgCHT1 protein (Lane 12) and flow through samples. One gel was silver stained and a replicate gel was used for Western immunoblotting, probed with mouse anti-chitinase active site peptide (B1993) polyclonal sera. Lanes 2 and 6 shows that the chitin beads pulled down the chitinases and interacting high molecular chitinase and protein complex band; washing with PBST did not detach the complex from the chitin beads. The duplex band (Lane 2 and 6, around 50 kDa, bottom panel) shows the with pro- and mature forms of PgCHT1 and two other higher immuno-reactive bands (100 kDa and just below 200 kDa) are homo or heteromeric forms of the chitinase-containing complex.
