# Supplementary material for: A Hetero-Multimeric Chitinase-Containing Plasmodium falciparum and Plasmodium gallinaceum Ookinete-Secreted Protein Complex Involved in Mosquito Midgut Invasion
Source: Front Cell Infect Microbiol. 2021 Jan 8;10:615343. doi: 10.3389/fcimb.2020.615343 (PMC7821095; doi:10.3389/fcimb.2020.615343)
Supplement: Supplementary Figure 4 — Serum containing media used for P. falciparum ookinete culture to affinity pull-down the P. falciparum chitinase complex. [file DataSheet_4.pdf]

Western blot analysis of affinity pulldown of *Plasmodium falciparum* chitinase complex from ookinete culture supernatants (M199 media used for ookinete culture contained 10% human serum).

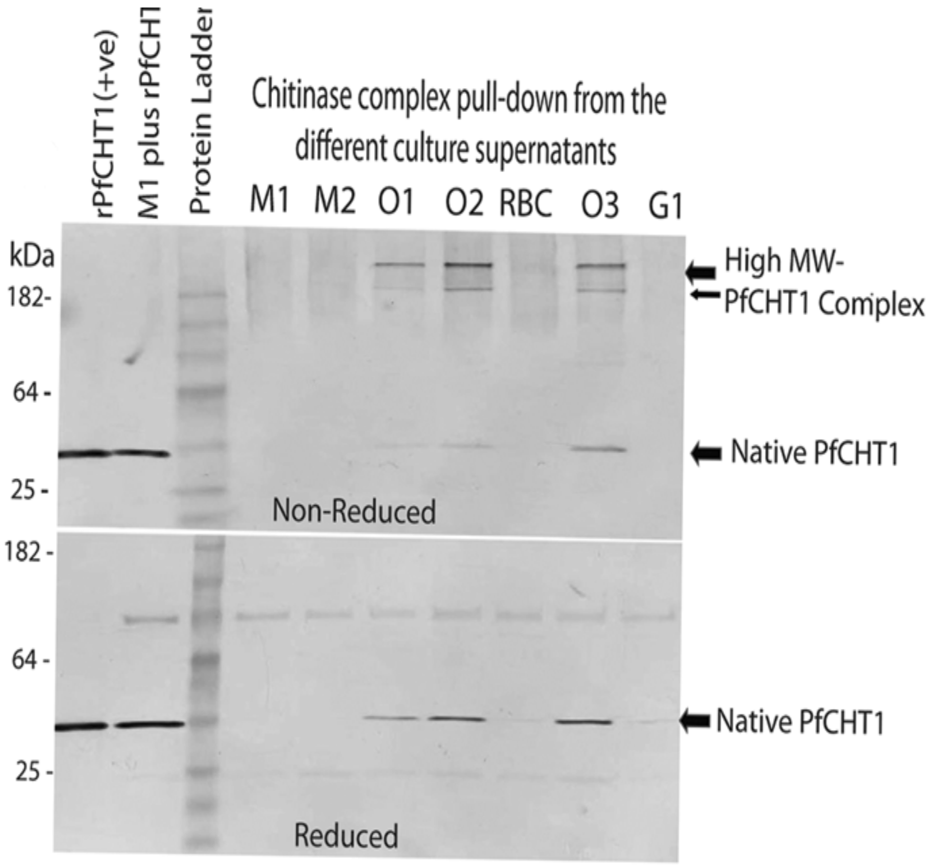

Supplemental Figure 4. Serum containing media used for *P. falciparum* ookinete culture to affinity pulldown of *P. falciparum* chitinase complex. Chitin beads were added to *P. falciparum* ookinete culture supernatant containing 10% human serum in triplicate (O1, O2 and O3) to affinity pull down chitinase-associated protein complexes from the ookinete secretome. Protein bound beads were washed three times with PBST, SDS sample buffer (non-reduced and reduced) added to the beads, then the material was boiled and supernatant analyzed by SDS-PAGE and Western immunoblot using anti-chitinase mAb (1C3). Samples were run under non-reducing (Top panel) or reducing conditions (bottom panel). The negative controls using chitin beads incubated in ookinete media alone (M1 and M2), lysed red blood cells in media (RBC), and mature gametocyte culture supernatant (G) and none of them showed positive bands. Recombinant PfCHT1 protein was used as a positive control. Both serum-containing and serum-free medium (Figure 4) showed high molecular chitinase complexes in non-reduced samples.
