# Supplementary material for: A Hetero-Multimeric Chitinase-Containing Plasmodium falciparum and Plasmodium gallinaceum Ookinete-Secreted Protein Complex Involved in Mosquito Midgut Invasion
Source: Front Cell Infect Microbiol. 2021 Jan 8;10:615343. doi: 10.3389/fcimb.2020.615343 (PMC7821095; doi:10.3389/fcimb.2020.615343)
Supplement: Supplementary Table 1 — List of primers used for generating transgenic P. berghei chimeric parasite lines expressing PfCHT1 and their product sizes. [file DataSheet_5.pdf]

Supplementary Table 1: List of primers (A) used for generating transgenic *P. berghei* chimeric parasite line expressing *PfCHT1* and product size (B)

A

Primer code	Sequence	Enzymes	Product (bp)	Description
**Primers used to generate construct pL2321**				
9450	ACTGGGCCCGTAAGGGCGTAAAAATAACCAAAGG	*ApaI*	1049	Forward *Pbcht1* HR1
9451	GAGCCGCGGTTTTTCTAAATTTTGTAAGTATATTAA	*SacII*		Reverse *Pbcht1* HR1
9452	CGAGGTACCGGATAAATACAAATATTTCTCACA	*KpnI*	973	Forward *Pbcht1* HR2
9453	CATGCGGCCGCTATTATTTAACGATGTTTTAGTTACAAAAAC	*NotI*		Reverse *Pbcht1* HR2
**Primers used to generate construct pL2322**				
9454	GAGCCGCGGATGAATTTTACTGTGAAATATTCATTTTTAG	*SacII*	1137	Forward *Pfcht1* ORF
9455	CGAGGTACCTTAATGTAAAGATTCTACGAAATATTC	*KpnI*		Reverse *Pfcht1* ORF
**Primers used for genotyping *PbΔcht1* (3152cl1)**				
9489	GTTGTAAAATTTTAATAGTTCATACCATTCACC		1316	5′ integration
7289	TAAAGCACAATATCTAGGATACTAC			
7922	GTCTCTTCAATGATTCATAAATAGTTGG		1320	3′ integration
9490	TATTATGAAAATGGTGAATTGACGCTTTAACC			
4698	GTTCGCTAAACTGCATCGTC		1108	SM
4699	GTTTGAGGTAGCAAGTAGACG			
9532	ATCCCTATCACATGTTGACGCACTCTGC		1506	*PbΔcht1* confirmation
9533	CTTGTTACCCATATAGCATCATTGTGTTTAGC			
**Primers used for genotyping *Pb-PfCHT1*(r) (3165cl1)**				
9454	GAGCCGCGGATGAATTTTACTGTGAAATATTCATTTTTAG		1155	*Pfcht1* ORF confirmation
9455	CGAGGTACCTTAATGTAAAGATTCTACGAAATATTC			
9489	GTTGTAAAATTTTAATAGTTCATACCATTCACC		1281	5′ integration
9536	CTCGATGTCCCTCAATGACACTTAC			
9537	TGTTTCCTTCAACCCCTTTTAATCCGAATGA		1369	3′ integration
9490	TATTATGAAAATGGTGAATTGACGCTTTAACC			
4698	GTTCGCTAAACTGCATCGTC		1108	SM
4699	GTTTGAGGTAGCAAGTAGACG			

B

Template	Product	Primers	Amplicon size
***PbΔcht1* (3152cl1)**	5’int	9489 & 7289	1316 bp
	3’int	7922 & 9490	1320 bp
	SM	4698 & 4699	1108 bp
	ORF	9530 & 9531	1506 bp
**Pb-PfCHT1 (r) (3165cl1)**	ORF	9454 & 9455	1155 bp
	5’int	9489 & 9536	1281bp
	3’int	9537 & 9490	1369bp
	SM	4698 & 4699	1108bp
